# Metabolomics reveals biomarkers of opioid use disorder

**DOI:** 10.1038/s41398-021-01228-7

**Published:** 2021-02-04

**Authors:** Reza Ghanbari, Yuanyuan Li, Wimal Pathmasiri, Susan McRitchie, Arash Etemadi, Jonathan D. Pollock, Hossein Poustchi, Afarin Rahimi-Movaghar, Masoumeh Amin-Esmaeili, Gholamreza Roshandel, Amaneh Shayanrad, Behrouz Abaei, Reza Malekzadeh, Susan C. J. Sumner

**Affiliations:** 1 Department of Nutrition, Nutrition Research Institute, University of North Carolina at Chapel Hill, Chapel Hill, NC USA; 2 Digestive Oncology Research Center, Digestive Diseases Research Institute, Tehran University of Medical Science, Tehran, Iran; 3 Division of Cancer Epidemiology and Genetics, National Cancer Institute (NCI), Bethesda, MD USA; 4 Genetics, Epigenetics, and Developmental Neuroscience Branch, National Institute on Drug Abuse (NIDA), Bethesda, MD USA; 5 Iranian National Center for Addiction Studies (INCAS), Tehran University of Medical Sciences (TUMS), Tehran, Iran; 6 Department of Mental Health, Bloomberg School of Public Health, Johns Hopkins University, Baltimore, MD USA; 7 Golestan Research Center of Gastroenterology and Hepatology, Golestan University of Medical Sciences, Gorgan, Iran

**Keywords:** Diagnostic markers, Scientific community

## Abstract

Opioid use disorder (OUD) is diagnosed using the qualitative criteria defined by the Diagnostic and Statistical Manual of Mental Disorders, Fifth Edition (DSM-5). Diagnostic biomarkers for OUD do not currently exist. Our study focused on developing objective biological markers to differentiate chronic opiate users with OUD from chronic opiate users without OUD. Using biospecimens from the Golestan Cohort Study, we compared the metabolomics profiles of high opium users who were diagnosed as OUD positive with high opium users who were diagnosed as OUD negative. High opium use was defined as maximum weekly opium usage greater than or equal to the median usage (2.4 g per week), and OUD was defined as having 2 or more DSM-5 criteria in any 12-month period. Among the 218 high opium users in this study, 80 were diagnosed as OUD negative, while 138 were diagnosed as OUD positive. Seven hundred and twelve peaks differentiated high opium users diagnosed as OUD positive from high opium users diagnosed as OUD negative. Stepwise logistic regression modeling of subject characteristics data together with the 712 differentiating peaks revealed a signature that is 95% predictive of an OUD positive diagnosis, a significant (*p* < 0.0001) improvement over a 63% accurate prediction based on subject characteristic data for these samples. These results suggest that a metabolic profile can be used to predict an OUD positive diagnosis.

## Introduction

More than fifty years have passed since Dole and Nyswander described opioid addiction as a metabolic disease, suggesting that opioids disrupt homeostasis to produce drug-seeking behavior in the face of adverse consequences^1^. An important issue in the addiction is that people exposed to opioids may develop dependence, but not Opioid Use Disorder (OUD)^2^. OUD is a chronic recurrent disorder that increasingly causes undesirable emotional states by involving the brain’s reward system and could include impaired social functioning^3,4^.

Despite significant advances in the genetics and neurobiology of addiction as a brain disease, and preliminary studies to discover biomarkers of OUD, validated systemic biomarkers for OUD do not exist^5^. Differential diagnosis of OUD is obtained through interview or questionnaire to determine if the patients meet the DSM-5 diagnostic criteria. These criteria include impaired control, social impairment, risky use, tolerance, withdrawal, craving, and continued use despite problems. Having at least two of the 11 criteria meets the diagnoses of OUD with the number of criteria met as an indicator of the severity of the OUD^6^.

Iran is a country with a high rate of opiate use. Opium is the main opiate used^7^. Our study focused on a random sample of opium users in the Golestan Cohort Study (GCS) in Iran, where more than 8400 individuals (about 17% of the participants) reported chronic opiate use with a median duration of use of 19 years^8^. 75% of the opium users in the full cohort used a combination of teriak and shireh with only 4 people reporting heroin use^8^.

We investigated urinary metabolomic profiles to reveal biomarkers that could differentiate high opium users who were diagnosed as OUD positive from high opium users who were diagnosed as OUD negative. Our investigation is important because optimized treatment relies on accurate diagnosis of OUD. The DSM-5, the predominant diagnostic instrument in psychiatry, has known limitations for diagnosis of substance abuse disorders^9^. Objective biological markers can improve the diagnosis that is currently based on subjective DSM-5 questionnaire. In addition metabolites that are increased or decreased in opium users diagnosed with OUD (compared with opium users not diagnosed with OUD) can be used to determine pathway perturbations, and lead to the identification of druggable or nutritional targets.

## Materials and methods

### Study population

The details of the GCS (a cohort of over 50,000 adults aged 40–75 living in Golestan Province, Northeast Iran) have been previously published^10^. The GCS was approved by appropriate ethics committees at Tehran University of Medical Sciences, the US National Cancer Institute (NCI, IRB# 07-C-N120), and the International Agency for Research on Cancer (IARC).

In 2018, a random sample of 451 GCS participants who reported long-term opium use and 92 never-users were recalled. They underwent a detailed interview using modified Persian and Turkman versions of the Section L of WHO Composite International Diagnostic Interview (CIDI, version 2.1) to diagnose lifetime OUD^7^, based on the Diagnostic and Statistical Manual of Mental Disorders, 5th edition (DSM‐5). The presence of 2 or more of the 11 criteria during any 12-month period of life was defined as lifetime OUD.

Variables considered for adjustment of the logistic regression models (see below) included age at enrollment, gender, tobacco use (current/former/never), BMI, and route of opium use (ingestion/inhalation). OUD diagnosis was the outcome for the models. Alcohol use was not included in the analysis, because it was rare in this population, and only 3.5% of participants reported ever using alcohol.

No subjects participated in drug-related treatment for addiction as part of the GCS. Cohort participants gave non-fasted spot urine samples which were stored at −20 °C until 2015 when they were transferred on dry ice to the NCI Biorepository and stored at −80 °C. Aliquots were then shipped to UNC Chapel Hill.

### Sample selection

In this sample selection, we excluded individuals who had discordant reports of their opiate use compared with baseline (baseline users who reported no lifetime opium use at the recall visit and vice versa, *n* = 24), and those without a urine sample available (*n* = 8). We also restricted the current analysis to high opium users reporting equal to or more than the median intake (2.4 g per week), to reduce the chance of misclassification. The final sample used in the current study included 138 urine samples from high opium users who were diagnosed as OUD positive, and 80 urine samples from high opium users diagnosed as OUD negative. Urine samples were selected from an additional 80 subjects who reported that they had never used opium.

### Untargeted metabolomics via ultra-performance liquid chromatography (UPLC) high-resolution mass spectrometry

Details of the sample preparation, data acquisition, data preprocessing and metabolite identification and annotation are provided in the Supplementary Material Section. In brief, urine samples were prepared according to published methods^11^, and untargeted metabolomics data were acquired on a Vanquish UHPLC systems coupled with a Q Exactive™ HF-X Hybrid Quadrupole-Orbitrap™ Mass Spectrometer (UPLC-HR-MS; Thermo Fisher Scientific). Data were processed using Progenesis QI (Waters Corporation). Peaks detected by UPLC-HR-MS were identified or annotated. Signals detected on our untargeted platform are matched to an in-house physical standards library that was developed by acquiring data for over 2000 chemical standards run under the same conditions to the study samples. The evidence basis for metabolite identifications and annotations are based on matching to our in-house library physical standards library (Ontology Level, OL), as well as to Public Databases (PD), and are detailed in the supplementary material.

### Hypothesis testing

Statistical tests for the normalized peaks in the metabolomics profiles were conducted using a two-tailed *t*-test with the Satterthwaite correction for unequal variances or the chi-square test. Statistical analyses were conducted using SAS 9.4 (SAS Institute Inc., Cary, NC). In this exploratory metabolomics study, *p*-values were not adjusted for multiple testing^12,13^. The nominal *p*-values are reported for the following comparisons 80 high opium users diagnosed as OUD negative versus 138 high opium users diagnosed as OUD positive.

### Logistic regression modeling

Logistic regression was used to model which peaks/metabolites were predictive of a positive OUD diagnosis. Several modeling approaches were used that included all normalized metabolomics peaks, or included subsets of peaks. Stepwise logistic regression procedures (criteria: model entry *p* < 0.1 and model removal *p* > 0.05) with standardization of continuous variables, was used for model selection. The Hosmer-Lemeshow goodness-of-fit test was used to assess the final model for adequacy. Receiver operating characteristics (ROC) curve and the area under the curve (AUC) were used to evaluate metabolites as predictors of OUD. Stepwise models were conducted, with and without subject characteristics as potential covariates, using:

- 712 peaks which differentiated high opium users who were diagnosed as OUD positive from high opium users who were diagnosed as OUD negative.
- 40 identified/annotated metabolites that differentiated the high opium users who were diagnosed as OUD positive from high opium users who were diagnosed as OUD negative (26 of these 40 metabolites also differentiated opium users from non-opium users).
- 14 identified/annotated metabolites that were unique to differentiation of high opium users who were diagnosed as OUD positive from high opium users who were diagnosed as OUD negative (but did not also differentiate opium users from non-opium users).

### Pathway enrichment: high opium users who were diagnosed as OUD positive from the high opium users who were diagnosed OUD as negative

Pathway enrichment was conducted using the Mummichog software in Metaboanalyst 4.0^14^. All features (*m*/*z*) remaining after filtering data were entered together with the *p*-value that was calculated for the comparison of high opium users who were diagnosed as OUD positive and high opium users who were diagnosed OUD as negative. A *p*-value cut-off of 0.01 was used to determine the size of the permutation group that the algorithm used for selecting significant features to match for all possible metabolites. A mass accuracy of 3 ppm was used as the threshold for annotations used in identifying candidate pathways. All possible metabolites which were matched by *m*/*z* were searched in the human reference metabolic network (hsa_*m*_*fn*), and the null distribution of module activities was estimated by using 100 permutations of random lists drawn from the experimental reference feature list. The candidate pathways were based on the similarity of *m*/*z*.

## Results

### Sample characteristics

The subject characteristics for the 218 high Opium users who were diagnosed as OUD positive (138 subjects) or OUD negative (80 subjects) are provided in Table 1. For these study samples, the OUD diagnosis was associated at *p* < 0.1 with age at the time of enrollment (*p* = 0.029, OUD positive were 2 years younger than OUD negative), and route of opium exposure (*p* = 0.054, higher by inhalation than by ingestion), but was not associated with BMI, gender, or tobacco use.

**Table 1:** Subject characteristics of high opium users diagnosed as OUD positive and high opium users diagnosed as OUD negative.

Characteristic	OUD positive (*n* = 138)	OUD negative (*n* = 80)	*p*-value^2^
Age at enrollment (yrs), mean (SD) [range]	49.0 (6.1) [39.7, 67.5]	51.0 (6.6) [40.5, 68.6]	**0.029**
Male (count, %)	110 (79.7%)	62 (77.5%)	0.700
Tobacco smoking status			0.141
Current smoker (count, %)	76 (55.1%)	37 (46.2%)	
Former smoker (count, %)	11 (8.0%)	13 (16.3%)	
Never smoker (count, %)	51 (36.9%)	30 (37.5%)	
Opium use, maximum nokhods/week, mean (SD) [range]^1^	31.0 (16.8) [12.0, 105.0]	29.8 (23.8) [12.0, 168.0]	0.698
Ever used alcohol			0.790
Yes	35 (25.4%)	19 (23.8%)	
No	103 (74.6%)	61 (76.3%)	
Body mass index, mean (SD) [range]	23.5 (4.2) [15.5, 37.5]	24.2 (4.7) [15.6, 37.3]	0.230
Route of opium administration			**0.054**
Inhalation	73 (52.9%)	53 (66.3%)	
Ingestion	65 (47.1%)	27 (33.7%)	
Severity of opioid use disorder, DSM-5			
Absent	0	80 (100%)	
Mild	65 (47.1%)	0	
Moderate	43 (31.2%)	0	
Severe	30 (21.7%)	0	

^1^Nokhod is the local measurement for the amount of opium used, and is equivalent to approximately 0.2 grams (*42*). The sample of 218 opium users was selected from 430 opium users with the following distribution of maximum nokhods per week: 119 subjects had low opium use (0.3–3.0), 93 subjects had moderate opium use (3.5–10.5), and 218 subjects had high opium use (≥ 12.0).

^2^Bold values indicate statistical significance *P* < 0.1.

### Metabolic profiles of high opium user diagnosed as OUD positive versus high opium user diagnosed as OUD negative

**Fig. 1:**
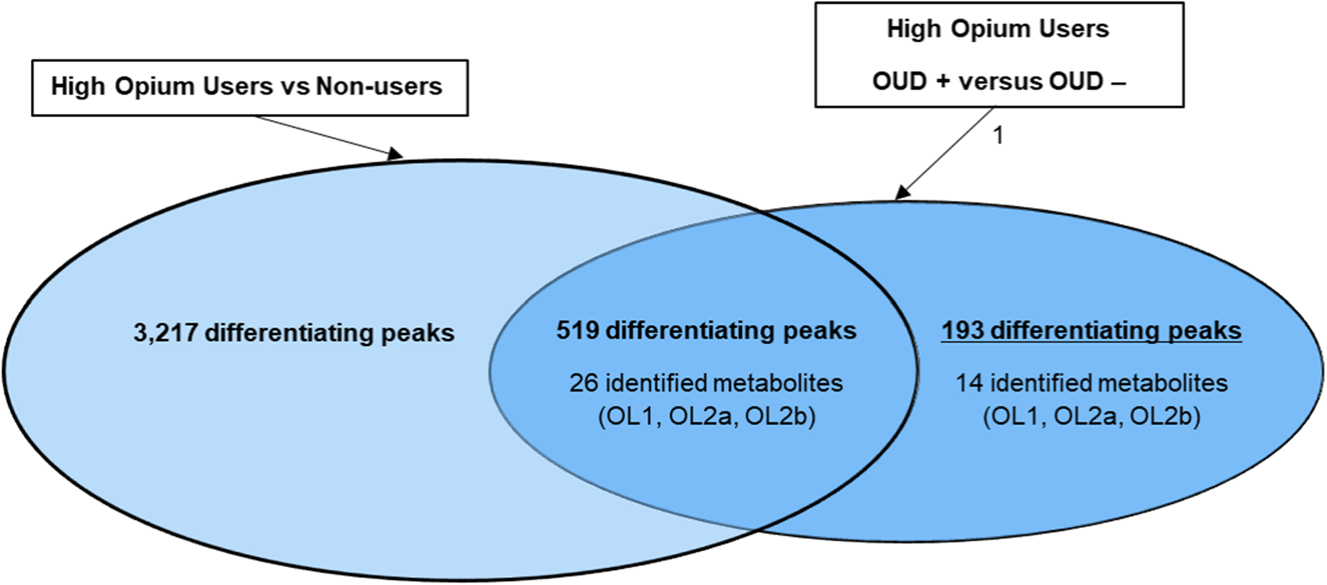
Over 3700 peaks differentiated high opium users from non-opium users. 712 peaks differentiated high opium users diagnosed as OUD positive from high opium users diagnosed as OUD negative. 193 peaks were unique to the differentiation OUD positive versus OUD negative high opium users. Metabolites were identified or annotated using an in-house physical standards library, and peaks were annotated using big data analytics.

Over 7714 UPLC-HRMS signals were obtained after data preprocessing. Hypothesis testing and fold change were determined for the normalized peaks in the metabolomics data set for the comparison of (a) high opium users diagnosed as OUD positive vs high opium users diagnosed as OUD negative, and (b) opium users vs non-opium users. Over 700 peaks (712) tested different by *t*-test (*p* < 0.10) between high opium users diagnosed as OUD positive versus high opium users diagnosed as OUD negative (Fig. 1). Forty of the 712 peaks were identified or annotated through matching to the in-house physical standards library (Table 2), while additional peaks were annotated using big data analytics (Table S1).

**Table 2:** Signals that differentiated (*p* < 0.10) high OUD positive opium users from high OUD negative opium users (Fig. 1) matched to 40 metabolites in the in-house physical standards library.

Ontology	Metabolite	*p*-Value	Fold change	Derivation^a^
OL1^b^	**Pterine**	0.001	1.3 (+)^c^	Vitamin B9 metabolism
OL2A	Deoxyadenosine	0.002	1.6 (+)	Purine metabolism
OL1	Morphine-6-beta-D-glucuronide	0.002	1.6 (+)	Opium use
OL1	Morphine-3-beta-D-glucuronide	0.004	1.6 (+)	Opium use
OL2B	Naloxone-3-beta-D-glucuronide	0.005	2.4 (+)	Opium use
OL1	Morphine	0.005	1.5 (+)	Opium use
OL2B	Octopamine	0.006	1.5 (+)	Tryptophan metabolism
OL1	Cotinine	0.006	1.6 (+)	Tobacco use
OL1	Codeine	0.007	1.4 (+)	Opium use
OL1	Codeine-6-beta-D-glucuronide	0.013	1.4 (+)	Opium use
OL2B	Morphine-3-beta-D-glucuronide	0.015	1.4 (+)	Opium use
OL2B	Serine	0.017	1.7 (−)	Amino acid metabolism
OL2B	Morphine	0.019	1.5 (+)	Opium use
OL2A	**6-Carboxyhexonate**	0.021	1.3 (−)	Fatty acid metabolism
OL2A	**N-Acetyl-S-(3,4-dihydroxybutyl)-L-cysteine**	0.023	3.5 (−)	Butadiene and acrylamide
OL1	**Sarcosine**	0.026	1.2 (+)	Amino acid methylation
OL2A	Codeine isomer or derivative	0.030	1.4 (+)	Opium use
OL1	Hydroxycotinine	0.034	1.4 (+)	Tobacco use
OL1	N-Acetyl-DL-tryptophan	0.035	1.2 (−)	Amino acid acetylation
OL2B	**Tryptophan**	0.038	1.3 (−)	Tryptophan metabolism
OL1	Codeine	0.040	1.3 (+)	Opium use
OL2B	Dihydromorphine	0.042	1.5 (+)	Opium use
OL1	N-Acetylcystine	0.048	1.1 (−)	Amino acid acetylation
OL1	**Mono-isobutyl phthalate**	0.049	2.1 (+)	Environmental exposure
OL1	**N-Methyl-D-aspartic acid**	0.049	1.2 (+)	Amino acid methylation
OL2B	**Lauroylcarnitine**	0.057	1.9 (−)	Carnitine metabolism
OL2A	Kynurenine	0.060	1.4 (−)	Tryptophan metabolism
OL1	Nicotine	0.068	1.5 (+)	Tobacco use
OL2B	N-Acetylproline	0.069	1.3 (−)	Amino acid acetylation
OL1	**2,4-Dihydroxypteridine**	0.069	1.2 (+)	Vitamin B9 metabolism
OL1	**Azelate**	0.071	1.4 (−)	Fatty acid oxidation
OL2B	N-Acetylcysteine	0.072	1.2 (−)	Amino acid acetylation
OL2A	Creatinine	0.079	1.1 (−)	Amino acid metabolism
OL2A	N-Acetylproline	0.081	1.1 (−)	Amino acid acetylation
OL2B	**Glycocholate**	0.082	1.4 (+)	Bile acid metabolism
OL1	N-Acetyl-S-(2-carbamoylethyl)-L-cysteine	0.084	1.2 (−)	Butadiene and acrylamide
OL2B	**Mono ethyl hexyl phthalate**	0.085	1.4 (+)	Environmental exposure
OL2B	**3-Methylhistamine**	0.087	1.4 (−)	Amino acid methylation
OL2A	N-Acetylphenylalanine	0.094	1.2 (−)	Amino acid acetylation
OL1	**Phosphorylcholine**	0.096	1.9 (+)	Choline metabolism

Fourteen of these metabolites (bold) were unique to the differentiation (*p* < 0.10) of high opium users who were diagnosed as OUD positive versus OUD negative.

^a^Derivation: Metabolites were derived from endogenous metabolism, opium use, tobacco use, or environmentally related exposure.

^b^Ontology: OL1, highly confident identification based on matching with in-house physical standard library (IPSL) via retention time (RT, with RT error ≤|0.5| min), exact mass (MS, with mass error <5 ppm), and tandem mass similarity based on experimental fragmentation spectra (experimental MS/MS, with similarity ≥30); OL2a, confident identification based on matching with IPSL via MS and RT; OL2b, annotation for the isomer or derivatives of the compound listed, based on matching with IPSL via MS and MS/MS.

^c^Direction of change. +/−, increased/decreased in OUD positive.

### Pathway enrichment

**Fig. 2:**
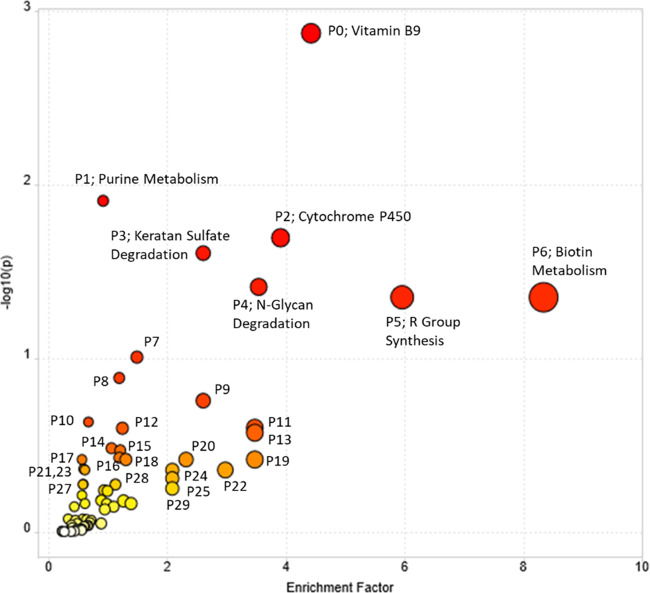
The distribution plot of the Enrichment Factor versus −log10(P) of the pathway enrichment analysis. Mummichog was used to evaluate the pathway enrichment of all features (*m*/*z*) with *p* < 0.01 based on the *t*-test for the comparison of high opium users who were diagnosed as OUD positive versus high opium users who were diagnosed OUD as negative was used as the threshold to determine the size of the permutation groups used by the algorithm.

Pathway enrichment was conducted in Metaboanalyst^14^ using all 7714 peaks. A cut-off for pathway significance (*p* < 0.01) was used to determine the size of the permutation group that the algorithm used to determine the enrichment between high opium users diagnosed as OUD positive versus high opium users diagnosed as OUD negative. The candidate pathways based on the match of exact mass (<3 ppm) of key metabolites that are included in the known pathway map are provided in Table S2. The distribution plot of the Enrichment Factor versus −log10 (*P*) is shown in Fig. 2. High opium users diagnosed as OUD positive versus those diagnosed as OUD negative had an enrichment for pathways involving biotin (vitamin B7), folate (vitamin B9), cytochrome P450 metabolism, purine metabolism, keratan sulfate degradation, N-glycan degradation, and R group synthesis. *Vitamin* absorption, bioavailability, and utilization are known to be impacted by drug addiction^15^. *Cytochrome P450s* are involved in the metabolism of opium, and the slow versus fast metabolism has been associated with addiction^16^. Opioid use has been shown to alter *purine* metabolism^17^. *Keratan sulfate* is a glycosaminoglycan that is at significant levels in central and peripheral nervous systems^18^. *N-glycan* is required to express the correctly folded form of the delta-opioid receptor^19^. R group synthesis is associated with the FAD/FADH2 conversion of fatty acids.

#### Modeling approach 1

Stepwise logistic regression was used to determine which of the 712 peaks were predictive of an OUD positive diagnosis. First, the area under the ROC curve (AUC) was calculated using the subject characteristics (Table 1) of age at the time of enrollment and route of opium use. This base model resulted in an AUC of 0.625 (Figure S1a). Second, all 712 peaks that differentiated (*p* < 0.10) the high opium users diagnosed as OUD positive from high opium users diagnosed as OUD negative was modeled without including subject characteristics. This resulted in an AUC of 0.720 which was significantly different (*p* = 0.042) from the base model. Third, all 712 peaks that differentiated (*p* < 0.10) the high opium users diagnosed as OUD positive from high opium users diagnosed as OUD negative was modeled with age at the time of enrollment and opium use as covariates. This resulted in an AUC of 0.946, which was significantly increased (*p* < 0.0001) over the base model. Using this modeling approach, only 16 peaks were selected that were predictive of an OUD positive diagnosis (Table 3). Two of the 16 peaks matched to pterin (OL1) and tryptophan (OL2b) using the in-house physical standards library. Annotations using public databases are provided for 6 additional peaks, while 8 of the peaks remained unknown unknowns.

**Table 3:** Metabolites predictive of a positive OUD diagnosis in high opium users.

Ontology^a^	Model 1^b^	Model 2^b^	Model 3
	*16 peaks* (Table S3)	*8 metabolites* (Table S4)	*5 metabolites* (Table S5)
OL1	Pterine (+)^c^	Pterine (+)	Pterine (+)
OL1	–	Sarcosine (+)	Sarcosine (+)
OL2b	Tryptophan (−)	Tryptophan (−)	Tryptophan (−)
OL1	–	Azelate (−)	Azelate (−)
OL2b	–	N-acetylproline (−)	–
OL2b	–	Octopamine (+)	–
OL2b	–	Serine (−)	–
OL1	–	Nicotine (+)	–
OL2a	–		N-Acetyl-dihydroxybutyl-cysteine (−)
PDd^d^	2-Polyprenyl-3-methyl-5-hydroxy-6-methoxy-1,4-benzoquinone	–	–
PDa	5α-androst-16-en-3α-ol	–	–
PDa	L-Tyrosinamide	–	–
PDb	D-1-[(3-Carboxypropyl)amino]-1-deoxyfructose	–	–
PDc	Alanyl-Proline	–	–
PDc	Phlorisobutyrophenone 2-glucoside	–	–

Age at time of enrollment and route of opium use were covariates in the model. Model 1 used 712 peaks that differentiated high opium users diagnosed as OUD positive from high opium users diagnosed as OUD negative. Models 2 and 3 included peaks matched using the in-house physical standards library.

^a^Ontology: OL1, highly confident identification based on matching with in-house physical standard library (IPSL) via retention time (RT, with RT error ≤|0.5| min), exact mass (MS, with mass error <5 ppm), and tandem mass similarity based on experimental fragmentation spectra (experimental MS/MS, with similarity ≥30); OL2a, confident identification based on matching with IPSL via MS and RT; OL2b, annotation for the isomer or derivatives of the compound listed, based on matching with IPSL via MS and experimental MS/MS.

^b^Eight of the 16 peaks that predicted OUD under Model 1 were identified or annotated, while 8 peaks (not listed) remained unknown.

^c^Direction of change. +/−, increased/decreased in OUD positive.

^d^PD, Public Data Base. PDa, annotation based on matching with PD via MS and experimental MS/MS (could be the listed compound, or the isomer or derivatives of the listed compound); PDb, annotation based on matching with public database via MS and predict MS/MS; PDc, annotation for the listed compound based on matching with public database via MS and isotopic similarity or adducts; PDd, annotation for the listed compound based on matching with public database via MS.

#### Modeling approach 2

Forty of the 712 peaks that differentiated (*p* < 0.10) high opium users diagnosed as OUD positive from high opium users diagnosed as OUD negative could be matched to the in-house physical standards library (Table 2, Fig. 1). Major differentiators included metabolites derived from opium use, tobacco use, involved in biopterins and vitamin B9, tryptophan metabolism, acetylation of amino acids, bile acids, fatty acids, and carnitine metabolism. In addition, *N*-Acetyl-S-(3,4-dihydroxybutyl)-L-cysteine (*p* = 0.023) and *N*-Acetyl-S-(2-carbamoylethyl)-L-cysteine (*p* = 0.083) were lower in urine of high opium users diagnosed as OUD positive vs high opium users diagnosed as OUD negative. N-Acetyl-S-(3,4-dihydroxybutyl)-L-cysteine and N-Acetyl-S-(2-carbamoylethyl)-L-cysteine (*p* = 0.083) are metabolic products of butadiene^20^ (BD) and acrylamide^21^ (AM), respectively. These metabolites have previously been detected in biospecimens from tobacco users at significantly higher levels than non-tobacco users. They are attributed to the metabolism of the parent compounds (AM and BD) that form during the curation process, or on combustion of tobacco^22^. They could also be formed in the curing and combustion of opium or other plant material.

Stepwise logistic regression using these 40 metabolites resulted in an AUC of 0.76, which was significantly increased (*p* = 0.0049) over the base model (Figure S1b). Including subject characteristics of age at the time of enrollment and route of opium exposure resulted in an AUC of 0.80, also significantly (*p* < 0.0001) increased from the base model. Metabolites that were predictive of an OUD positive diagnosis (Table 3) included tryptophan, pterine, sarcosine, N-acetylproline, azelate, octopamine, serine, and nicotine.

### Metabolic profiles unique to the OUD positive versus OUD negative diagnosis

Five hundred and nineteen of the 712 peaks that tested different between OUD positive high opium users versus high OUD negative high opium users, also differentiated the opium users from non-opium users (Fig. 1). To provide a focus on only metabolites that are important to the diagnosis of OUD, the 519 signals that were also important to differentiation of opium users from non-opium users were excluded for this analysis. This resulted in 193 peaks unique to the differentiation (*p* < 0.10) of subjects diagnoses as OUD positive versus those diagnoses as OUD negative. Of these 193 peaks, only 14 peaks that defined the OUD diagnosis matched to the in-house physical standards library. These 14 metabolites are listed in Table 2, while the additional annotated peaks through public databases are provided in Table S1.

Eleven of the 14 peaks that matched to the in-house library that were most important to defining OUD included the following endogenous metabolites: pterine (*p* = 0.0011), 2,4-dihydroxypterine (*p* = 0.0695), sarcosine (*p* = 0.0263), phosphorylcholine (*p* = 0.0962), 6-carboxyhexonate (*p* = 0.021), lauroylcarnitine (0.0574), glycocholate (*p* = 0.0816), 3-methylhistamine (0.087), azelate (*p* = 0.0713), n-methyl-D-aspartic acid (*p* = 0.0488), and tryptophan (0.0378).

The biological significance of these 11 endogenous metabolites is summarized:

- *Pterin* is part of biopterin and folate. Biopterins are cofactors for aromatic amino acid hydroxylases, which are involved in the synthesis of dopamine, norepinephrine, epinephrine, and serotonin, and trace amines^23^. The active form of folate (vitamin B9) is tetrahydrofolate which accepts and donates one carbon unit (methyl group). *Dihydroxypteridine* is involved in folate and riboflavin pathways^24^.
- *Phosphorylcholine* is derived from phosphorylation of choline^25^, and *sarcosine* is an intermediate in the metabolism of choline to glycine^26^.
- *N-Methyl-d-aspartic acid* (NMDA) is an agonist at the NMDA receptor and mimics the action of glutamate^27^, and *tryptophan* is in the neurotransmitter pathway^28^. *Azelaic acid* (AZA) is a competitive inhibitor of tyrosinase in vitro^29^.
- *Lauroylcarnitine* is associated with fatty oxidation disorders involving acyl CoA dehydrogenase deficiency, and carnitine palmitoyltransferase I and II deficiency^30^. *6-Carboxyhexanoic acid* is a medium-chain fatty acid derived from heptanedioic acid and is involved in the gut microbial biosynthesis of biotin^31^.
- *Glycocholate* is a secondary bile acid, produced in the microbial flora of the colonic environment by bacteria^32^, and is absorbed and recirculated. Bile acids are important for absorption of hydrophobic nutrients, dietary fats and vitamins, and the regulation enzymes involved in cholesterol homeostasis.
- *3-Methylhistamine* is a prominent metabolite of histamine, which has a role in allergy, inflammation, gastric acid secretion, and neurotransmission^33^.

Three metabolites (Table 2) derived from exogenous exposures were also important to the differentiation of the high opium users who were diagnosed as OUD positive from high opium users who were diagnosed as OUD negative. These included mono-isobutyl phthalate (*p* = 0.0485, +) and mono ethyl hexyl phthalate (*p* = 0.0852, +), which could arise as metabolic products following ingestion of phthalates that leach from plastics used in inhalation of opium. N-Acetyl-S-(3,4-dihydroxybutyl)-L-cysteine was also a differentiator (*p* = 0.0228, −), and is presumably derived as a metabolic product of BD intake associated with the curing or combustion of plant matter.

#### Modeling approach 3

Stepwise logistic regression using the 14 metabolites unique to the differentiation of OUD positive versus OUD negative, together with covariates of age at the time of enrollment and route of opium use resulted in an AUC of 0.751, which was significantly increased (*p* < 0.0005) over the base model (Figure S1c). Stepwise logistic regression using only the 14 metabolites (with no subject characteristics) resulted in an ACU of 0.706, which was not significantly increased (*p* = 0.127) over the base model. Results from Model 3 (with or without the covariates of age at time of enrollment and route of opium use) show 5 of the 14 metabolites (pterine, sarcosine, tryptophan, azelate, and N-Acetyl-S-(3,4-dihydroxybutyl)-L-cysteine) as predictive of a positive OUD (Table 3).

## Discussion

Our study revealed metabolomics signatures of OUD in a predominantly Turkmen population of chronic high opium users. We provide metabolite identifications and annotations for 712 features detected using untargeted mass spectrometry that are important to the differentiation of high opium users diagnosed as OUD positive from high opium users diagnosed as OUD negative. None of these identifications are known metabolites derived from other drugs of abuse. Pathway enrichment analysis points to a general disruption in vitamin B9 (folate), vitamin B7 (biotin), cytochrome P450, purine, and glycan metabolism, and FAD/FADH2 conversion of fatty acids.

Stepwise logistic regression analysis of these 712 peaks, together with subject characteristics, resulted in 16 candidate peaks that predict 95% of the high opium users who were diagnosed as OUD positive.

Forty of the 712 features which differentiate high opium users who were diagnosed as OUD positive from high opium users who were diagnosed as OUD negative matched to an in-house physical standards library. Models constructed with only these 40 metabolites predicted 80% of the subjects diagnosed as OUD positive, selecting 8 metabolites as predictors. Predictors of an OUD diagnosis in these high opium users included an increase in three endogenous compounds (pterine, sarcosine, and octopamine), a decrease in four endogenous compounds (tryptophan, azelate, N-acetylproline, and serine), and an increase in nicotine. Fourteen metabolites were determined to be unique to OUD diagnosis, after subtracting analytes known to overlap with opium use. Models using these 14 metabolites predicted 75% of the subjects testing OUD positive and replicated pterine, sarcosine, tryptophan, and azelate as metabolite predictors.

Many identified or annotated metabolites that differentiated high opium users who were OUD positive from high opium users who were OUD negative play a significant role in neurotransmitter synthesis and signal transduction^34^. Tryptophan is the major amino acid precursor of serotonin (5HT). 5HT deficits have been implicated in physical symptoms and emotional dysphoria following withdrawal from opioids^35^. Alterations in sarcosine, serine, kyneurate, NMDA found in this study are consistent with the observations that glutamatergic signaling is disrupted by opioids^36^. Sarcosine (methyl-glycine) acts as an NMDA receptor agonist and a glycine receptor agonist^37^. Serine is converted to D-serine by serine racemace. D-serine acts as co-agonist with glutamate to activate NMDA receptors^38^. Kyneurate a metabolite of tryptophan metabolized to quinolinic acid acts as a NMDA receptor agonist^39^. Octopamine is a trace amine that is an agonist of TAAR1 receptors implicated in mediating the actions of drugs of abuse^40^. Methylhistamine is a histamine receptor (H3) agonist that inhibits the firing of cholinergic neurons in the ventral striatum and decreases dopamine release^41^.

Limitations of the current study include the (a) use of self-report for the amount of opium consumed, (b) assumption that symptoms over any 12-month period-of-time are accurately recalled, (c) estimated amount/grams of opium may vary within or among regions, and be underestimated^42^, (d) analyses were not stratified by the route of administration or type of opiate used due to the sample size, and because this paper focuses on a marker of OUD independent of route, (e) that the current study was not powered for multiple testing, and (f) that the metabolomic profiles are not quantitated, or replicated in this cohort or across cohorts.

The year that the individuals met a DSM-5 OUD diagnosis during their history of opium use is unknown because the DSM-5 interview was not conducted at the time of baseline urine collection. These urinary baseline metabolomic profiles presented herein could result from chronic opium use, and/or from inherent individual metabolic differences present prior to the acquisition of OUD.

Chronic use of opiates and opioids without meeting the criteria for DSM-5 OUD is not unique to the Turkman population for opiate use. Some chronic pain patients treated with prescription opioids and chronic users of illicit opioids do not meet the criteria for DSM-5 OUD. This suggests that the approaches used in this study are likely to be generalizable to other cohorts. This is also consistent with other substance use disorders where heavy use does not necessarily imply a substance used disorder^43^.

Research on biomarkers for OUD and other substance use disorders has focused on neuroimaging (MRI, fMRI, and PET) and EEG studies^44^. While these biomarkers may eventually be clinically validated in other populations, they will be costly to implement. In contrast, validation of biomarkers in other populations in accessible biological fluids (e.g., urine, blood, saliva) will be less costly, and easier to implement in general medical practice. In addition, these non-invasive biomarkers will be important complements to results from neuroimaging studies.

In conclusion, if the current results are replicated, the identification of peripheral biomarkers for OUD would represent a significant advancement in defining and managing the disease. It would further validate the Dole and Nyswander hypothesis that OUD is a brain disease in which metabolism is disrupted, and would provide biomarkers for OUD that could be used to optimize treatment. In addition, validation of the discovered metabolic perturbations related to vitamins and fatty acids could lead to the development of a nutrient cocktail to test in clinical settings for efficacy to mitigate symptoms that lead to the diagnosis of OUD.

## Supplementary information

SUPPLEMENTARY MATERIAL

Figure S1
