# Supplementary material for: Metabolomics reveals biomarkers of opioid use disorder
Source: Transl Psychiatry. 2021 Feb 4;11:103. doi: 10.1038/s41398-021-01228-7 (PMC7862627; doi:10.1038/s41398-021-01228-7)
Supplement: Supplementary file 1 — SUPPLEMENTARY MATERIAL [file 41398_2021_1228_MOESM1_ESM.docx]

**SUPPLEMENTARY MATERIAL**

**Metabolomics Reveals Biomarkers of Opioid Use Disorder**

Reza Ghanbari^1,2,#^ , Yuanyuan Li^1,#^, Wimal Pathmasiri^1^, Susan McRitchie^1^, Arash Etemadi^3^, Jonathan D. Pollock^4^, Hossein Poustchi^2^, Afarin Rahimi-Movaghar^5^, Masoomeh Amin-Esmaeili^5,6^, Gholamreza Roshandel^7^, Amaneh Shayanrad^2^, Behrouz Abaei^2^, Reza Malekzadeh^2,^*, Susan CJ Sumner^1,*^

1. Department of Nutrition, Nutrition Research Institute, University of North Carolina at Chapel Hill, Chapel Hill, NC, USA
2. Digestive Oncology Research Center, Digestive Diseases Research Institute, Tehran University of Medical Science, Tehran, Iran
3. Division of Cancer Epidemiology and Genetics, National Cancer Institute (NCI), Bethesda, MD, USA
4. Genetics, Epigenetics, and Developmental Neuroscience Branch, National Institute on Drug Abuse (NIDA), Bethesda, MD, USA
5. Iranian National Center for Addiction Studies (INCAS), Tehran University of Medical (TUM) Sciences, Tehran, Iran
6. Department of Mental Health, Bloomberg School of Public Health, Johns Hopkins University, Baltimore, MD, USA
7. Golestan Research Center of Gastroenterology and Hepatology, Golestan University of Medical Sciences, Gorgan, Iran

# Co-first authors

*Corresponding Author for Epidemiology and Clinical Assessments

Reza Malekzadeh, MD/PhD

Distinguished Professor of Medicine

Digestive Oncology Research Center, Digestive Diseases Research Institute

Tehran University of Medical Science, Iran

Tel: 0+98-21-82415106

Email. [dr.reza.malekzadeh@gmail.com](mailto:dr.reza.malekzadeh@gmail.com)

*Corresponding Author for Metabolomics, Pathways, Biochemical Interpretations, Modelling

Susan CJ Sumner, PhD

Professor, Department of Nutrition

Nutrition Research Institute

University of North Carolina at Chapel Hill

Chapel Hill, NC, 27514, USA

Tel: 919-622-4456

Email: [Susan_sumner@unc.edu](mailto:Susan_sumner@unc.edu)

**Checklist of Supplementary Material:**

**1). Sample preparation, data acquisition, data preprocessing and metabolite identification and annotation for untargeted metabolomics via UPLC high resolution mass spectrometry.**

**2). Table S1. Signals that differentiated high opium users diagnosed as OUD positive from high opium users diagnosed as OUD negative that were identified or annotated using the in-house physical standards library or annotated using public databases.**

**3). Table S2. Candidate pathways, based on the similarity of m/z using Mummichog, that differentiated high opium users diagnosed as OUD positive from high opium users diagnosed as OUD negative.**

**4). Table S3. Logistic Modeling Results for Model 1 including demographics and untargeted metabolomics data (712 peaks that differentiated high opium users diagnosed as OUD positive from high opium users diagnosed as OUD negative were used as potential predictors)**

**5). Table S4. Logistic Modeling Results for Model 2 including demographics and untargeted metabolomics data (40 identified/annotated peaks that differentiated high opium users diagnosed as OUD positive from high opium users diagnosed as OUD negative were used as potential predictors)**

**6). Table S5. Logistic Modeling Results for Model 3 including demographics and untargeted metabolomics data (14 identified/annotated metabolites that were unique to the differentiation of high opium users diagnosed as OUD positive from high opium users diagnosed as OUD negative (but did not also differentiate opium users from non-opium users) were used as potential predictors)**

**7). Figure S1: Comparison of AUC for candidate peaks/metabolites and subject characteristic covariates. Logistic regression models using a) 712 peaks that differentiate high opium users diagnosed as OUD positive verse high opium users diagnosed as OUD negative, b) 40 metabolites that differentiate high opium users diagnosed as OUD positive versus high opium users diagnosed as OUD negative, or c) 14 metabolites unique to the diagnosis of OUD.**

**1). Sample preparation, data acquisition, data preprocessing and metabolite identification and annotation for untargeted metabolomics via UPLC high resolution mass spectrometry.**

Urine samples were prepared according to the published methods (10), with modification. In brief, 50-µL of urine sample was mixed with 400-µL methanol containing 500 ng/ml L-tryptophan-d5, and vortex at 5,000 rpm for 2 min. Quality control samples (QC pool) were prepared by pooling 7-µL urine from each of the study samples and processed identically to the study samples. Study samples and QC pools were centrifuged at 16, 000 rcf for 5 min at 4°C. The supernatant (320-µL) was dried and reconstituted with 100 µl water-methanol (95:5, v/v). The study samples were randomized with interspersed QC pools before data acquisition.

Metabolomics data was acquired on a Vanquish UHPLC systems coupled with a Q Exactive™ HF-X Hybrid Quadrupole-Orbitrap™ Mass Spectrometer (UPLC-HR-MS; Thermo Fisher Scientific, San Jose, CA). Metabolites were separated via an HSS T3 C18 column (2.1 x 100 mm, 1.7 µm, Waters Corporation) at 50 °C with binary mobile phases, which are water (A) and methanol (B), each containing 0.1% formic acid (v/v). The UHPLC linear gradient started from 2% B, and increased to 100% B in 16 min, then held for 4 min, with a flow rate at 0.4 ml/min. The untargeted data was collected from 70 to 1050 m/z under the data dependent acquisition (DDA) mode.

The untargeted data was processed by Progenesis QI (version 2.1, Waters Corporation) for peak picking, alignment, and normalization. The highly varied signal (peak) with RSD>30% across QC pools, and the signal with missing value in all QC pools were excluded for further analysis. Signals that highly varied (RSD>30%) or that were missing across the QC Pools were excluded for further analysis. If any missing values remain then Progenesis imputes a value. Peaks were normalized in Progenesis QI using the “normalize to total intensity” feature.

Metabolite Identification and Annotation: Peaks detected by UPLC-HR-MS were identified or annotated through matching to an in-house experimental standards library generated by acquiring data for over 1,000 compounds under identical conditions to the study samples, as well as to public database (including HMDB, METLIN, and NIST). Identifications and annotations used available data for retention time (RT), exact mass (MS), MS/MS fragmentation pattern, and isotopic pattern. Signals/metabolites reported in the results and discussion section that matched to the in-house experimental standards library by (a) RT, MS, and MS/MS are labeled as OL1, or (b) by RT and MS are labeled OL2a. An OL2b label was provided for signals that match by MS and MS/MS to the in-house library, that were outside the retention time tolerance (±0.5 min) for the standards run under identical conditions. Signals matched to public databases are labeled as PDa (MS and experimental MS/MS), PDb (MS and theoretical MS/MS), PDc (MS and isotopic similarity or adducts), and PDd (MS only) are also provided in supplemental material.

**2). Supplementary** **Table S1. Signals that differentiated high opium users diagnosed as OUD positive from high opium users diagnosed as OUD negative that were identified or annotated using the in-house physical standards library or annotated using public databases**

| **Metabolite/Peak**  **(712 signals, 351 with annotation or identification)**^1^ |  | **Ontology level**^2^ | **FC**^3^ | ***p*-value**^4^ |
| --- | --- | --- | --- | --- |
| Phosphorylcholine |  | OL1 | 1.9 | 0.096 |
| L-Glutamic acid |  | OL1 | 1.2 | 0.049 |
| Sarcosine |  | OL1 | 1.2 | 0.026 |
| Trans-3'-hydroxycotinine | x | OL1 | 1.4 | 0.034 |
| Nicotine | x | OL1 | 1.5 | 0.068 |
| Pterine |  | OL1 | 1.3 | 0.001 |
| Monoisobutyl phthalic acid |  | OL1 | 2.1 | 0.049 |
| Cotinine | x | OL1 | 1.6 | 0.006 |
| 2,4-dihydroxypteridine |  | OL1 | 1.2 | 0.070 |
| N-acetylcysteine | x | OL1 | -1.1 | 0.048 |
| N-Acetyl-S-(carbamoylethyl) -L-cysteine | x | OL1 | -1.2 | 0.084 |
| Morphine-3-beta-D-glucuronide | x | OL1 | 1.6 | 0.004 |
| Morphine | x | OL1 | 1.5 | 0.005 |
| Morphine-6-beta-D-glucuronide | x | OL1 | 1.6 | 0.002 |
| Codeine | x | OL1 | 1.4 | 0.007 |
| Codeine-6-beta-D-glucuronide | x | OL1 | 1.4 | 0.013 |
| Codeine | x | OL1 | 1.3 | 0.040 |
| N-Acetyl-DL-tryptophan | x | OL1 | -1.2 | 0.035 |
| Azelate |  | OL1 | -1.4 | 0.071 |
| Creatinine | x | OL2A | -1.1 | 0.079 |
| Kynurenine | x | OL2A | -1.4 | 0.060 |
| N-Acetyl-S-(3,4-dihydroxybutyl) -L-cysteine |  | OL2A | -3.5 | 0.023 |
| N-acetylproline | x | OL2A | -1.1 | 0.081 |
| Codeine | x | OL2A | 1.4 | 0.030 |
| 6-carboxyhexanoate |  | OL2A | -1.3 | 0.021 |
| Syringic acid | x | OL2A | -1.2 | 0.049 |
| N-acetylphenylalanine | x | OL2A | -1.2 | 0.094 |
| Morphine-3-beta-D-glucuronide | x | OL2B | 1.4 | 0.015 |
| 3-methylhistamine |  | OL2B | -1.4 | 0.087 |
| P-octopamine | x | OL2B | 1.5 | 0.006 |
| Glycocholate |  | OL2B | 1.4 | 0.082 |
| Lauroylcarnitine |  | OL2B | -1.9 | 0.057 |
| Monoethylhexyl phthalic acid |  | OL2B | 1.4 | 0.085 |
| L-serine | x | OL2B | -1.7 | 0.017 |
| Dihydromorphine | x | OL2B | 1.5 | 0.042 |
| N-acetylcysteine | x | OL2B | -1.2 | 0.072 |
| Morphine | x | OL2B | 1.5 | 0.019 |
| N-acetylproline | x | OL2B | -1.3 | 0.069 |
| Naloxone-3-beta-D-glucuronide | x | OL2B | 2.4 | 0.005 |
| Tryptophan |  | OL2B | -1.3 | 0.038 |
| Glycerophosphocholine | x | PDa | 1.2 | 0.047 |
| Pro-thr-ser |  | PDa | -1.3 | 0.074 |
| 6-hydroxypseudooxynicotine | x | PDa | 1.4 | 0.020 |
| 2-pyridylethanol | x | PDa | 1.5 | 0.066 |
| Decanoyl-L-carnitine |  | PDa | -1.6 | 0.037 |
| Decanoyl-L-carnitine | x | PDa | -1.6 | 0.022 |
| Caryophyllene epoxide |  | PDa | -1.2 | 0.078 |
| 1,2-Dihexanoyl-sn-glycerol | x | PDa | -1.3 | 0.094 |
| Dodecanedioic acid | x | PDa | -1.9 | 0.088 |
| 5-Pregnen-3β-ol-20-one | x | PDa | 1.4 | 0.098 |
| 5α-androst-16-en-3α-ol | x | PDa | -1.7 | 0.033 |
| N-Acetyl-L-glutamic acid | x | PDa | -1.1 | 0.049 |
| 2',4'-dihydroxyacetophenone | x | PDa | 1.2 | 0.078 |
| Asp-Gly-Val |  | PDa | -1.5 | 0.076 |
| L-Theanine | x | PDa | -1.2 | 0.093 |
| Glu-Val | x | PDa | -1.1 | 0.098 |
| N-lactoyl-phenylalanine | x | PDa | 1.5 | 0.097 |
| 2-deoxy-2,3-dehydro-N-acetylneuraminic acid | x | PDa | 1.6 | 0.002 |
| His-Val | x | PDa | -1.3 | 0.094 |
| L-Tyrosinamide | x | PDa | 1.2 | 0.056 |
| 1,3-Dimethyluric acid | x | PDa | 1.8 | 0.085 |
| P-Acetaminobenzoic acid | x | PDa | 1.3 | 0.036 |
| 4',7-Dimethoxy-8-methylisoflavone | x | PDa | 1.9 | 0.018 |
| Acetyl-L-Tyrosine |  | PDa | -1.4 | 0.083 |
| 2'-Hydroxy-3-methoxychalcone | x | PDa | 1.9 | 0.004 |
| 2'-Methoxy-6-methylflavone | x | PDa | 1.9 | 0.002 |
| 3-Phenoxypropionic acid | x | PDa | 1.2 | 0.013 |
| Gamma-hexalactone | x | PDa | -1.1 | 0.073 |
| Ser-Asn-Thr |  | PDa | -1.8 | 0.068 |
| Salicylic Acid -β-D-O-Glucuronide | x | PDa | 5.0 | 0.045 |
| 5-hydroxyferulate | x | PDa | 2.7 | 0.083 |
| O-acetylsalicylhydroxamic acid | x | PDa | 2.5 | 0.086 |
| Ile-Arg-Phe |  | PDa | -2.3 | 0.084 |
| 4-Methyl-3-phenylcoumarin | x | PDa | 2.0 | 0.008 |
| L-yrosinamide | x | PDa | 1.4 | 0.041 |
| S-Allyl-L-cysteine |  | PDa | -2.1 | 0.075 |
| 2'-Hydroxy-3-methoxychalcone | x | PDa | 1.8 | 0.017 |
| 3-(3,4-Dimethoxyphenyl)-4-methylcoumarin | x | PDa | 1.5 | 0.079 |
| 3'-hydroxyflavone | x | PDa | 1.7 | 0.091 |
| KAPA (8-Amino-7-oxononanoic acid) | x | PDa | 1.6 | 0.091 |
| 3-phenylcoumarin | x | PDa | 1.7 | 0.060 |
| Noscapine | x | PDa | 1.7 | 0.052 |
| 1-Naphthoic acid | x | PDa | 1.4 | 0.082 |
| Mandelic acid |  | PDa | 2.8 | 0.053 |
| 2,6-dihydroxynaphthalene |  | PDa | 17.4 | 0.054 |
| 20-HETE Ethanolamide | x | PDa | 1.4 | 0.072 |
| A-L-threo-4-Hex-4-enopyranuronosyl-D-galacturonic acid | x | PDb | -1.3 | 0.046 |
| N-butylformamide | x | PDb | 1.2 | 0.023 |
| Gamma-glutamyl-Hydroxyproline |  | PDb | 1.5 | 0.072 |
| 6-[3-(benzoyloxy)-2-methoxy-6-(prop-2-en-1-yl)phenoxy]-3,4,5-trihydroxyoxane-2-carboxylic acid | x | PDb | 1.7 | 0.010 |
| Diphenol glucuronide |  | PDb | 1.2 | 0.021 |
| Porphobilinogen | x | PDb | 1.5 | 0.035 |
| Dopamine 4-sulfate | x | PDb | 1.7 | 0.003 |
| Tyramine-O-sulfate | x | PDb | 1.3 | 0.080 |
| 2-Mercaptopropanoic acid | x | PDb | 1.6 | 0.055 |
| Dopamine 4-sulfate | x | PDb | 1.5 | 0.006 |
| Muramic acid |  | PDb | -2.2 | 0.075 |
| 2,6 Dimethylheptanoyl carnitine | x | PDb | -1.4 | 0.025 |
| Glucosyl (E)-2,6-Dimethyl-2,5-heptadienoate | x | PDb | -1.2 | 0.091 |
| Moracin H |  | PDb | 7.3 | 0.056 |
| Riesling acetal |  | PDb | -2.1 | 0.046 |
| N-(1-Deoxy-1-fructosyl) methionine |  | PDb | 1.3 | 0.086 |
| Cucurbic acid | x | PDb | -1.6 | 0.045 |
| (S)-3-Octanol glucoside | x | PDb | -1.2 | 0.077 |
| 3-hydroxyundecanoyl carnitine | x | PDb | -1.4 | 0.051 |
| Taraxacolide 1-O-b-D-glucopyranoside | x | PDb | 1.2 | 0.032 |
| Betaxolol | x | PDb | -1.3 | 0.097 |
| 4,8 Dimethylnonanoyl carnitine |  | PDb | -1.9 | 0.039 |
| 12-Ketodeoxycholic acid |  | PDb | 1.4 | 0.082 |
| Cholic acid glucuronide |  | PDb | 1.7 | 0.044 |
| Riesling acetal | x | PDb | -1.9 | 0.045 |
| 4-hydroxy ketorolac |  | PDb | 34.1 | 0.087 |
| Glycochenodeoxycholic acid 3-glucuronide | x | PDb | 1.7 | 0.028 |
| (3a,5b,7a,12a)-24-[(carboxymethyl)amino]-1,12-dihydroxy-24-oxocholan-3-yl-b-D-Glucopyranosiduronic acid | x | PDb | 1.5 | 0.040 |
| Eremopetasidione |  | PDb | 1.2 | 0.055 |
| Deoxycholic acid 3-glucuronide |  | PDb | 1.3 | 0.090 |
| (Z)-3-Oxo-2-(2-pentenyl)-1-cyclopenteneacetic acid | x | PDb | 1.2 | 0.095 |
| 4'-Methylliquiritigenin 7-rhamnoside | x | PDb | 1.4 | 0.007 |
| Naproxen O-glucuronide | x | PDb | -1.9 | 0.050 |
| Tyramine-O-sulfate | x | PDb | 1.5 | 0.030 |
| Hydromorphone-3-sulphate | x | PDb | 1.6 | 0.017 |
| Agaritinal | x | PDb | -1.1 | 0.090 |
| Hydromorphone-3-glucoside | x | PDb | 1.2 | 0.096 |
| Toralactone | x | PDb | 1.3 | 0.026 |
| Isoalliin | x | PDb | -3.2 | 0.017 |
| Diphenol glucuronide | x | PDb | 1.9 | 0.047 |
| 3,4-Dihydroxy-tamoxifen | x | PDb | -1.7 | 0.089 |
| Voglibose | x | PDb | -1.2 | 0.077 |
| Hawkinsin | x | PDb | -1.2 | 0.078 |
| 4'-Hydroxy-R-phenprocoumon | x | PDb | 1.8 | 0.001 |
| 6-Hydroxy-5-methoxyindole glucuronide | x | PDb | 1.4 | 0.044 |
| D-1-[(3-Carboxypropyl) amino]-1-deoxyfructose |  | PDb | 1.2 | 0.071 |
| 6-[1-(2H-1,3-benzodioxol-5-yl)-3-(6-hydroxy-4-methoxy-1-benzofuran-5-yl)-1,3-dioxopropan-2-yl]-3,4,5-trihydroxyoxane-2-carboxylic acid | x | PDb | 1.8 | 0.006 |
| Narceinone | x | PDb | 1.6 | 0.015 |
| Prolylhydroxyproline |  | PDb | -1.3 | 0.086 |
| Succinyladenosine | x | PDb | -1.1 | 0.081 |
| Cassythicine | x | PDb | 1.7 | 0.004 |
| Esmolol | x | PDb | 1.3 | 0.056 |
| (2E)-3-(2-hydroxyphenyl)-1-(4-methoxyphenyl) prop-2-en-1-one | x | PDb | 1.5 | 0.099 |
| Nordihydroisomorphine | x | PDb | 2.1 | 0.024 |
| 6'-malonyltrifolirhizin | x | PDb | 1.9 | 0.001 |
| 3,4,5-trihydroxy-6-{[3,4,5-trihydroxy-5-(hydroxymethyl) oxolan-2-yl]methoxy}oxane-2-carboxylic acid |  | PDb | 1.7 | 0.011 |
| 3,4,5-trihydroxy-6-[(8-hydroxy-2-oxo-2H-chromen-7-yl) oxy]oxane-2-carboxylic acid | x | PDb | 1.2 | 0.068 |
| Benzisothiazole piperazine | x | PDb | 2.1 | 0.026 |
| 3,4,5-trihydroxy-6-(4-hydroxy-1H-indole-3-carbonyloxy) oxane-2-carboxylic acid | x | PDb | 1.5 | 0.046 |
| Trp asp | x | PDb | 1.4 | 0.039 |
| Hydromorphone-3-sulphate | x | PDb | 1.9 | 0.020 |
| (S)-nandigerine | x | PDb | 1.6 | 0.012 |
| 3,4,5-trihydroxy-6-{[7-methoxy-4-oxo-2-phenyl-8-(3,4,5-trihydroxyoxan-2-yl)-4H-chromen-5-yl]oxy}oxane-2-carboxylic acid | x | PDb | 2.1 | 0.023 |
| N-[2-(3,4-dimethoxyphenyl)ethyl]-3-[4-methoxy-3-(sulfooxy)phenyl]prop-2-enimidic acid | x | PDb | 1.7 | 0.008 |
| (4-ethenyl-2-hydroxy-6-methoxyphenyl) oxidanesulfonic acid | x | PDb | 1.3 | 0.046 |
| 2-aminoheptanoate | x | PDb | 1.2 | 0.084 |
| Triazolopropionic acid |  | PDb | -1.5 | 0.082 |
| 3,5-dihydroxy-8-(hydroxymethyl)-2-(4-hydroxyphenyl)-8-methyl-4H,8H-pyrano[2,3-f]chromen-4-one |  | PDb | 3.1 | 0.026 |
| Cysteinyl-serine | x | PDb | 2.6 | 0.002 |
| Juzirine | x | PDb | 2.1 | 0.001 |
| N-acetylcystathionine | x | PDb | 1.7 | 0.020 |
| 6-[5-(2-{[2-(3,4-dimethoxyphenyl)ethyl]-C-hydroxycarbonimidoyl}ethyl)-2-methoxyphenoxy]-3,4,5-trihydroxyoxane-2-carboxylic acid | x | PDb | 1.9 | 0.009 |
| 5'-(3'-Methoxy-4'-hydroxyphenyl)-gamma-valerolactone |  | PDb | 1.5 | 0.065 |
| 2,8-Dihydroxyquinoline-beta-D-glucuronide | x | PDb | 1.2 | 0.038 |
| Maysin | x | PDb | 1.9 | 0.036 |
| 6-[1-(3,4-dimethoxyphenyl)-2-{[3-(3,4-dimethoxyphenyl)-1-hydroxypropylidene]amino}ethoxy]-3,4,5-trihydroxyoxane-2-carboxylic acid | x | PDb | 2.7 | 0.007 |
| Acetaminophen glucuronide |  | PDb | 1.4 | 0.055 |
| 3,4,5-trihydroxy-6-[3-(4-methoxyphenyl)propoxy]oxane-2-carboxylic acid | x | PDb | 1.6 | 0.018 |
| 6-acetylmorphine | x | PDb | 1.5 | 0.082 |
| Chorismate | x | PDb | 2.0 | 0.031 |
| 6-{[1-(2-{[3-(3,4-dimethoxyphenyl)-1-hydroxypropylidene]amino}ethyl)-3-methoxy-4-oxocyclohexa-2,5-dien-1-yl]oxy}-3,4,5-trihydroxyoxane-2-carboxylic acid | x | PDb | 1.6 | 0.020 |
| 5-Hydroxy, 6-methoxy duloxetine sulfate | x | PDb | 1.9 | 0.042 |
| 1,3-diaminopropane | x | PDb | 1.6 | 0.012 |
| 6-Hydroxy-5-methoxyindole glucuronide | x | PDb | 1.4 | 0.042 |
| Oripavine | x | PDb | 2.3 | 0.017 |
| 3-(3,4-dimethoxyphenyl)-N-[2-(3,4-dimethoxyphenyl)ethyl]propanimidic acid | x | PDb | 2.1 | 0.010 |
| N-trans-Feruloyl-4-O-methyldopamine | x | PDb | 1.4 | 0.060 |
| 3,4,5-trihydroxy-6-{3-hydroxy-5-[(E)-2-(4-hydroxyphenyl)ethenyl]-2-[(1E)-3-methylbut-1-en-1-yl]phenoxy}oxane-2-carboxylic acid | x | PDb | 1.7 | 0.018 |
| 6-{[1-(2-{[3-(3,4-dimethoxyphenyl)-1-hydroxyprop-2-en-1-ylidene]amino}ethyl)-3-methoxy-4-oxocyclohexa-2,5-dien-1-yl]oxy}-3,4,5-trihydroxyoxane-2-carboxylic acid | x | PDb | 1.8 | 0.008 |
| Dihydroxycitracridone I | x | PDb | 2.0 | 0.030 |
| 7-Methoxy-2-methylisoflavone | x | PDb | 1.7 | 0.022 |
| (R)-glabridin | x | PDb | 1.6 | 0.036 |
| 6-acetylmorphine | x | PDb | 1.7 | 0.017 |
| Methyl 2,6-dihydroxy-4-quinolinecarboxylate | x | PDb | 1.9 | 0.020 |
| (E)-5-(3,4,5,6-Tetrahydro-3-pyridylidenemethyl)-2-furanmethanol | x | PDb | 1.8 | 0.005 |
| 6-beta-Naltrexol | x | PDb | 1.9 | 0.003 |
| 3,4,5-trihydroxy-6-{[3-hydroxy-2-(hydroxymethyl)-2-methylpropanoyl]oxy}oxane-2-carboxylic acid |  | PDb | 1.1 | 0.090 |
| 3,17-Dihydroxy-16-methoxytricyclo[12.3.1.12,6]nonadeca-1(17),2,4,6(19),14(18),15-hexaen-9-one | x | PDb | 2.3 | 0.009 |
| 6-{[3-(6,7-dimethoxy-2H-1,3-benzodioxol-5-yl)oxiran-2-yl]methoxy}-3,4,5-trihydroxyoxane-2-carboxylic acid |  | PDb | -1.5 | 0.061 |
| 6-{4-[(3,3-dimethyloxiran-2-yl)methyl]-3-hydroxyphenoxy}-3,4,5-trihydroxyoxane-2-carboxylic acid | x | PDb | 1.5 | 0.020 |
| Stavudine |  | PDb | -1.3 | 0.068 |
| Strobilurin A | x | PDb | 1.8 | 0.049 |
| Tryptophyl-proline |  | PDb | -7.9 | 0.053 |
| 7-(hydroxymethyl)-6-[(E)-2-methoxyethenyl]-2H-chromen-2-one |  | PDb | 6.0 | 0.054 |
| 2-(2H-1,3-benzodioxol-5-yl)-5-(3-hydroxypropyl)-1-benzofuran-7-ol |  | PDb | 11.7 | 0.089 |
| Medicarpin 3-O-(6'-malonylglucoside) |  | PDb | 24.3 | 0.078 |
| Pyrrolidonecarboxylic acid |  | PDc | 1.1 | 0.084 |
| Serylserine | x | PDc | 1.3 | 0.088 |
| 8-hydroxyguanine |  | PDc | 1.3 | 0.092 |
| Hydroxyprolyl-Leucine |  | PDc | -1.7 | 0.032 |
| Gly-Asp-Arg | x | PDc | 1.3 | 0.091 |
| Trans-3-Hydroxycotinine glucuronide | x | PDc | 1.6 | 0.023 |
| 1-Phenylpyrazolidin-3-one | x | PDc | 1.5 | 0.016 |
| Poppy acid | x | PDc | 2.1 | 0.007 |
| L-alpha-Amino-5-oxo-2(5H)-isoxazolepropanoic acid | x | PDc | -1.2 | 0.057 |
| 4-Hydroxyphenylacetylglutamic acid | x | PDc | 1.6 | 0.010 |
| Glutamyllysine | x | PDc | -1.2 | 0.076 |
| Asn-Ser |  | PDc | -1.4 | 0.019 |
| 2-(6-carboxy-3,4,5-trihydroxyoxan-2-yl)propanedioic acid | x | PDc | 1.7 | 0.001 |
| Argininic acid | x | PDc | 1.4 | 0.079 |
| Menadione |  | PDc | 4.1 | 0.009 |
| Cerulenin |  | PDc | 1.2 | 0.054 |
| Testosterone isocaproate | x | PDc | -1.5 | 0.067 |
| (-)-erythro-Anethole glycol 2-glucoside |  | PDc | 1.1 | 0.083 |
| Undecanedioic acid | x | PDc | -1.4 | 0.034 |
| Glucosyl (2E,6E,10x)-10,11-dihydroxy-2,6-farnesadienoate |  | PDc | -1.2 | 0.094 |
| Alpha-Ionol O-[arabinosyl-(1->6)-glucoside] |  | PDc | -2.0 | 0.022 |
| (6e,8e)-4,6,8-megastigmatriene |  | PDc | 1.2 | 0.072 |
| 7(14)-Bisabolene-2,3,10,11-tetrol |  | PDc | -1.3 | 0.080 |
| 3-dehydroquinate | x | PDc | 1.4 | 0.061 |
| N-Octanoyl-L-Homoserine lactone | x | PDc | 1.3 | 0.061 |
| 1,11-Undecanedicarboxylic acid | x | PDc | -2.1 | 0.055 |
| Tyr-Tyr-Arg |  | PDc | 1.5 | 0.073 |
| N-Phenylacetyl pyroglutamic acid |  | PDc | 3.7 | 0.092 |
| 4-hydroxy ketorolac |  | PDc | 37.2 | 0.097 |
| Malyngamide H |  | PDc | 1.4 | 0.026 |
| N-(3,4-Dimethylphenyl)-3-(3-(nitrophenyl)acrylamide | x | PDc | -1.6 | 0.045 |
| 3,7-Dimethyl-5-octene-1,7-diol 1-glucoside | x | PDc | 1.8 | 0.029 |
| Neryl Rhamnosyl-glucoside | x | PDc | 1.3 | 0.085 |
| Pro-His-Gln | x | PDc | 1.6 | 0.068 |
| Asn-lys-oh | x | PDc | 1.3 | 0.099 |
| 4-Guanidinobutyric acid |  | PDc | -1.2 | 0.034 |
| 2-Oxo-4-methylthiobutanoic acid | x | PDc | -2.6 | 0.016 |
| 7-methylhypoxanthine | x | PDc | 1.1 | 0.066 |
| Folinic acid | x | PDc | 1.4 | 0.032 |
| 5-methylthioribose |  | PDc | -4.5 | 0.069 |
| (1alpha,2alpha,4betah,6alpha,8R)-p-Menthane-2,6,8,9-tetrol | x | PDc | -1.3 | 0.095 |
| N-Acetylcystathionine | x | PDc | 1.3 | 0.015 |
| Pyroglutamic acid | x | PDc | -2.4 | 0.018 |
| Beta-carboline | x | PDc | 1.7 | 0.017 |
| 2-cyano-3-(3,4-dihydroxy-5-nitrophenyl)-N,N-diethylpropanamide | x | PDc | 1.6 | 0.016 |
| (1S,2S,4R,8R)-p-Menthane-1,2,8,9-tetrol | x | PDc | -1.5 | 0.091 |
| Histidinyl-Arginine | x | PDc | -1.3 | 0.081 |
| N-Carbamoyl-2-amino-2-(4-hydroxyphenyl)acetic acid |  | PDc | -1.5 | 0.056 |
| (2s,2's)-pyrosaccharopine |  | PDc | -1.3 | 0.034 |
| 2-pyrroloylglycine | x | PDc | -1.2 | 0.082 |
| 4-Hydroxy-5-phenyltetrahydro-1,3-oxazin-2-one | x | PDc | 1.2 | 0.069 |
| 2-[4-hydroxy-3-(sulfooxy)phenyl]acetic acid |  | PDc | 1.3 | 0.028 |
| N-Acetylserotonin sulfate |  | PDc | 1.4 | 0.070 |
| Narceinone | x | PDc | 1.9 | 0.004 |
| Narcotoline | x | PDc | 3.4 | 0.012 |
| N-(3-Oxooctanoyl)-L-homoserine lactone | x | PDc | -1.3 | 0.082 |
| (Z)-Narceine imide | x | PDc | 1.5 | 0.011 |
| N-Butyryl-L-homoserine lactone | x | PDc | -1.4 | 0.064 |
| 4-Hydroxy-alprenolol | x | PDc | 1.5 | 0.028 |
| 3-Ethyl-2-methoxypyrazine | x | PDc | -1.4 | 0.094 |
| Naloxone-3-beta-D-glucuronide | x | PDc | 1.7 | 0.008 |
| {4-[(1E)-3-oxobut-1-en-1-yl]phenyl}oxidanesulfonic acid | x | PDc | 1.6 | 0.072 |
| Amoxicillin |  | PDc | 1.8 | 0.012 |
| Alanyl-proline | x | PDc | -1.2 | 0.036 |
| Morphine | x | PDc | 2.2 | 0.006 |
| 1-piperidinecarboxaldehyde | x | PDc | 1.5 | 0.049 |
| Apo-[3-methylcrotonoyl-coa:carbon-dioxide ligase (ADP-forming)] | x | PDc | 1.4 | 0.057 |
| Pd 98059 | x | PDc | 2.2 | 0.011 |
| Trp-Trp-Asp | x | PDc | 1.9 | 0.002 |
| 3-Hydroxy-2H-pyran-2-one | x | PDc | 1.4 | 0.086 |
| Acetaminophen glucuronide | x | PDc | 1.4 | 0.000 |
| Tyr-Leu | x | PDc | -1.3 | 0.049 |
| 4-(2-Aminophenyl)-2,4-dioxobutanoic acid |  | PDc | 3.0 | 0.024 |
| Trp-Trp-Glu | x | PDc | 1.6 | 0.010 |
| Cartormin | x | PDc | 2.3 | 0.004 |
| 2-(2-Butoxyethoxy)acetic acid | x | PDc | 1.2 | 0.007 |
| 2-Methoxyacetaminophen glucuronide |  | PDc | 1.3 | 0.057 |
| 3,4,5-trihydroxy-6-(3-methoxyphenoxy)oxane-2-carboxylic acid |  | PDc | 1.6 | 0.031 |
| Margrapine A | x | PDc | 2.0 | 0.001 |
| Pilocarpine |  | PDc | -1.3 | 0.027 |
| L-Menthyl acetoacetate | x | PDc | 1.4 | 0.015 |
| Trans-4-(Aminomethyl)cyclohexanecarboxylic acid | x | PDc | 1.1 | 0.090 |
| Methyl dioxindole-3-acetate | x | PDc | -1.5 | 0.073 |
| Isobutyryl carnitine | x | PDc | 1.9 | 0.076 |
| N-cis-Caffeoyltyramine | x | PDc | 1.8 | 0.025 |
| 5-(3',4'-Dihydroxyphenyl)-gamma-valerolactone-3'-O-methyl-4'-O-glucuronide |  | PDc | 1.5 | 0.089 |
| Indole-3-carboxylic acid | x | PDc | 1.2 | 0.054 |
| Oxypurinol | x | PDc | -1.5 | 0.017 |
| Phlorisobutyrophenone 2-glucoside | x | PDc | -2.3 | 0.037 |
| Trp-Trp-Glu | x | PDc | 1.7 | 0.003 |
| 4'-Methyl-(-)-epigallocatechin 3-(4-methyl-gallate) | x | PDc | 1.4 | 0.091 |
| Cartormin | x | PDc | 2.7 | 0.019 |
| Methiocarb | x | PDc | 1.2 | 0.076 |
| (E)-5-(3,4,5,6-Tetrahydro-3-pyridylidenemethyl)-2-furanmethanol |  | PDc | 1.3 | 0.086 |
| 4-hydroxyvalsartan | x | PDc | 1.6 | 0.059 |
| 5,6-Isopropylidene-L-ascorbic acid | x | PDc | 1.8 | 0.010 |
| 3-methyldioxyindole | x | PDc | 1.3 | 0.069 |
| Herierin III |  | PDc | -1.2 | 0.050 |
| Lansamide 4 | x | PDc | 1.9 | 0.007 |
| Gly-Phe-Phe | x | PDc | 1.6 | 0.063 |
| Moracin L | x | PDc | 1.8 | 0.049 |
| [3,5-dihydroxy-2-(hydroxymethyl)-6-(2,4,6-trihydroxyphenyl)oxan-4-yl]oxidanesulfonic acid | x | PDc | 1.2 | 0.075 |
| 3,6-Ditigloyloxytropan-7-ol | x | PDc | 1.3 | 0.031 |
| Indoleacetyl glutamine | x | PDc | 1.8 | 0.018 |
| (R)-pelletierine | x | PDc | 1.8 | 0.083 |
| Alkaloid RC | x | PDc | 1.8 | 0.056 |
| Adenosine |  | PDc | 1.2 | 0.082 |
| (Â±)-hexanoylcarnitine | x | PDc | -1.2 | 0.036 |
| Harpagide |  | PDc | -1.3 | 0.036 |
| N-(-)-Jasmonoyl-(S)-threonine | x | PDc | 1.4 | 0.051 |
| 5-(2-Methylpropyl)tetrahydro-2-oxo-3-furancarboxylic acid |  | PDc | -1.3 | 0.083 |
| Oxynarcotine | x | PDc | 1.4 | 0.071 |
| Gibberellin A79 |  | PDc | 2.0 | 0.092 |
| Physangulide | x | PDc | 1.7 | 0.004 |
| Citramalate |  | PDc | -1.4 | 0.063 |
| Pteleine | x | PDc | 1.4 | 0.046 |
| (2E)-3-[3-(sulfooxy)phenyl]prop-2-enoic acid | x | PDc | -1.2 | 0.089 |
| 2-Polyprenyl-3-methyl-5-hydroxy-6-methoxy-1,4-benzoquinone | x | PDc | 1.7 | 0.014 |
| Asp-ala-oh |  | PDc | 4.1 | 0.077 |
| 5,7-Dimethoxy-4-methylcoumarin |  | PDc | 3.6 | 0.024 |
| 3,4,5-trihydroxy-6-{2-methoxy-4-[(5-oxooxolan-2-yl)methyl]phenoxy}oxane-2-carboxylic acid | x | PDc | 2.6 | 0.033 |
| Dihydroferuperine | x | PDc | 1.6 | 0.029 |
| 11-methoxyyangonin |  | PDc | -4.0 | 0.097 |
| D-1-Amino-2-pyrrolidinecarboxylic acid | x | PDd | 1.2 | 0.058 |
| Gamma-Glutamyl-S-methylcysteine sulfoxide | x | PDd | -1.2 | 0.043 |
| 3-(3-hydroxyphenyl)-2-phenyl-4-[(E)-2-phenylethenyl]-2,3-dihydro-1-benzofuran-6-ol | x | PDd | 1.4 | 0.026 |
| 5-[2H-Pyrrol-4-(3H)-ylidenemethyl]-2-furanmethanol | x | PDd | 1.2 | 0.034 |
| 2-Polyprenyl-3-methyl-5-hydroxy-6-methoxy-1,4-benzoquinone |  | PDd | 1.4 | 0.010 |
| Tetranor-PGDM | x | PDd | 1.9 | 0.060 |
| 2-amino-4-({1-[(carboxymethyl)-C-hydroxycarbonimidoyl]-2-[(2,3-dihydroxy-1-phenylpropyl)sulfanyl]ethyl}-C-hydroxycarbonimidoyl)butanoic acid |  | PDd | 1.4 | 0.055 |
| 2-amino-4-({1-[(carboxymethyl)-C-hydroxycarbonimidoyl]-2-[(2-hydroxy-2-methyl-3-oxo-1-phenylbutyl)sulfanyl]ethyl}-C-hydroxycarbonimidoyl)butanoic acid |  | PDd | 1.4 | 0.056 |
| Narceinone | x | PDd | 1.5 | 0.100 |
| 2-Isopropyl-5-methoxypyrazine | x | PDd | -1.3 | 0.090 |
| Cinnamyl cinnamate |  | PDd | -3.6 | 0.007 |
| Trans-3,4-Dihydro-3,4-dihydroxy-7,12-dimethylbenz[a]anthracene |  | PDd | -1.5 | 0.071 |
| Arginyl-methionine | x | PDd | 1.6 | 0.005 |
| Epoxiconazole |  | PDd | -1.2 | 0.088 |
| 4-[(E)-2-[6-hydroxy-3-(3-hydroxyphenyl)-2-phenyl-2,3-dihydro-1-benzofuran-4-yl]ethenyl]benzene-1,3-diol | x | PDd | 2.5 | 0.095 |
| N-Acetylserotonin sulfate | x | PDd | 1.4 | 0.074 |
| Albendazole sulfoxide | x | PDd | 2.1 | 0.095 |
| (Z)-1,3-Tridecadiene-5,7,9,11-tetrayne | x | PDd | 1.5 | 0.018 |
| 5-hydroxyoct-5-enoylglycine | x | PDd | -1.2 | 0.052 |
| Jasmolone glucoside | x | PDd | 2.0 | 0.002 |
| 1-(Methylsulfinyl)propyl 1-propenyl disulfide | x | PDd | 1.2 | 0.066 |
| Porric acid C | x | PDd | -1.3 | 0.026 |
| Kyotorphin | x | PDd | -1.4 | 0.026 |
| Blumealactone C |  | PDd | -1.7 | 0.042 |
| Ile asp leu arg |  | PDd | -2.0 | 0.056 |
| Anaxagoreine | x | PDd | 1.6 | 0.081 |
| 1-(3,5-dihydroxyphenyl)-3-phenylpropan-1-one | x | PDd | -1.4 | 0.068 |
| (R)-3-hydroxybutyrylcarnitine |  | PDd | -1.3 | 0.051 |
| 3,4,5-trihydroxy-6-[2-(3-hydroxyprop-1-en-1-yl)phenoxy]oxane-2-carboxylic acid |  | PDd | 1.6 | 0.090 |
| Ethanesulfonic acid, 2-[(2,6-diethylphenyl)(methoxymethyl)amino]-2-oxo- | x | PDd | 1.6 | 0.033 |
| 3-hydroxy-4-(3-hydroxyphenyl)-1-methyl-5-phenylpiperidine-2,6-dione | x | PDd | 1.6 | 0.033 |
| Pemirolast | x | PDd | -1.5 | 0.095 |
| Butylparaben |  | PDd | 1.1 | 0.073 |
| 3,6-dihydroxy-5-(3-hydroxyphenyl)-1-methyl-4-phenylpiperidin-2-one | x | PDd | 1.6 | 0.026 |
| Moracin G |  | PDd | 1.3 | 0.017 |
| Quinacrine | x | PDd | 1.5 | 0.024 |
| 7.71_372.1368n |  |  | -1.3 | 0.095 |
| 0.56_361.8037n |  |  | -1.4 | 0.013 |
| 0.65_114.0739m/z |  |  | -1.1 | 0.077 |
| 0.65_209.1124n | x |  | -1.1 | 0.053 |
| 0.65_227.1194n | x |  | -1.1 | 0.097 |
| 0.71_117.1103m/z | x |  | -1.1 | 0.097 |
| 0.75_191.1264n | x |  | 1.4 | 0.035 |
| 0.77_176.0661n | x |  | 1.5 | 0.015 |
| 0.84_154.0299n |  |  | -3.8 | 0.037 |
| 0.86_214.0617n |  |  | -2.1 | 0.035 |
| 0.86_348.1400m/z | x |  | -1.1 | 0.096 |
| 0.87_347.0962n | x |  | 1.9 | 0.047 |
| 0.89_103.0947m/z | x |  | 1.4 | 0.011 |
| 0.89_372.1143n | x |  | 1.3 | 0.095 |
| 1.01_108.0808m/z | x |  | -1.2 | 0.078 |
| 1.01_156.0457n | x |  | -1.1 | 0.100 |
| 1.01_190.0604n |  |  | -1.3 | 0.022 |
| 1.09_280.1169n |  |  | 1.7 | 0.086 |
| 1.15_193.0926n | x |  | 1.5 | 0.035 |
| 1.35_254.1826n | x |  | 2.2 | 0.067 |
| 1.37_163.1192n | x |  | 1.5 | 0.071 |
| 1.41_167.1058n | x |  | -1.6 | 0.054 |
| 1.47_224.0238m/z |  |  | -1.1 | 0.049 |
| 1.51_206.0117m/z |  |  | 1.1 | 0.094 |
| 1.51_347.1084m/z |  |  | 1.3 | 0.058 |
| 1.64_452.0856m/z | x |  | -1.3 | 0.096 |
| 1.87_183.1322m/z |  |  | -1.8 | 0.042 |
| 1.89_235.0311n | x |  | 1.7 | 0.005 |
| 1.89_387.1219m/z | x |  | 2.1 | 0.028 |
| 1.91_196.0406n | x |  | -2.9 | 0.013 |
| 10.08_284.1251n | x |  | -1.2 | 0.077 |
| 10.08_343.2389n | x |  | -1.4 | 0.059 |
| 10.08_360.1163n | x |  | -1.3 | 0.051 |
| 10.34_415.2025n | x |  | 1.6 | 0.033 |
| 10.39_374.1576n | x |  | 1.2 | 0.085 |
| 10.69_318.2551m/z |  |  | -2.0 | 0.046 |
| 10.71_360.1838m/z |  |  | 1.2 | 0.039 |
| 10.74_293.0951m/z | x |  | 1.5 | 0.093 |
| 10.76_371.2305n | x |  | 1.3 | 0.082 |
| 10.86_470.2265n | x |  | -1.7 | 0.022 |
| 10.93_233.1622n | x |  | -1.4 | 0.053 |
| 10.93_318.2549m/z | x |  | -1.7 | 0.032 |
| 10.98_407.2539m/z | x |  | -1.6 | 0.041 |
| 10.98_409.2694m/z | x |  | -1.8 | 0.016 |
| 11.06_463.2935n |  |  | 3.1 | 0.022 |
| 11.08_582.2655n |  |  | 1.3 | 0.041 |
| 11.20_368.0853m/z |  |  | -5.6 | 0.095 |
| 11.23_390.1893n |  |  | 1.3 | 0.077 |
| 11.28_346.2253n | x |  | -1.6 | 0.064 |
| 11.35_323.1964m/z |  |  | 1.4 | 0.089 |
| 11.40_250.1412m/z |  |  | 1.4 | 0.079 |
| 11.43_443.1976n | x |  | 1.2 | 0.086 |
| 11.48_381.1518m/z |  |  | 1.3 | 0.050 |
| 11.48_436.2296n |  |  | 1.5 | 0.007 |
| 11.60_383.2129n | x |  | 2.8 | 0.098 |
| 11.75_369.2053n |  |  | 2.2 | 0.072 |
| 11.80_269.1625n | x |  | -1.9 | 0.075 |
| 11.80_476.2280n | x |  | 1.3 | 0.089 |
| 11.82_436.2307n |  |  | 1.3 | 0.081 |
| 11.85_301.1322m/z | x |  | -1.9 | 0.056 |
| 11.87_460.2459n | x |  | -3.3 | 0.027 |
| 11.97_255.0845m/z |  |  | 49.7 | 0.087 |
| 11.97_309.0318n |  |  | 20.2 | 0.069 |
| 12.07_504.2634m/z |  |  | 1.9 | 0.098 |
| 12.07_527.2555n |  |  | 1.5 | 0.099 |
| 12.14_384.1759n | x |  | 1.4 | 0.079 |
| 12.29_383.1675m/z |  |  | 1.1 | 0.054 |
| 12.53_249.2086n |  |  | 1.5 | 0.038 |
| 12.53_418.2202n |  |  | 1.1 | 0.093 |
| 12.68_368.1889m/z |  |  | 1.4 | 0.067 |
| 12.70_333.6614m/z |  |  | 1.4 | 0.096 |
| 12.70_447.2983n |  |  | 1.2 | 0.090 |
| 12.90_595.3722n |  |  | 1.4 | 0.077 |
| 13.26_370.2045m/z |  |  | 1.6 | 0.049 |
| 13.46_529.2324m/z | x |  | 1.4 | 0.078 |
| 13.70_452.2022n | x |  | 1.2 | 0.074 |
| 2.00_263.1098m/z |  |  | 3.3 | 0.053 |
| 2.02_141.0884n | x |  | 1.2 | 0.050 |
| 2.14_177.0982n | x |  | 1.6 | 0.006 |
| 2.32_220.0041n | x |  | -1.3 | 0.099 |
| 2.32_276.9689m/z | x |  | -1.3 | 0.083 |
| 2.34_245.0898n | x |  | -1.3 | 0.010 |
| 2.36_224.1023n |  |  | -1.2 | 0.029 |
| 2.36_243.0712n | x |  | -1.3 | 0.001 |
| 2.36_285.0514m/z | x |  | -1.3 | 0.023 |
| 2.39_484.1341m/z | x |  | 1.6 | 0.004 |
| 2.39_96.5785m/z |  |  | -1.2 | 0.074 |
| 2.65_294.1811m/z |  |  | -1.6 | 0.074 |
| 2.69_195.0631m/z | x |  | -1.2 | 0.062 |
| 2.69_270.0398m/z | x |  | 1.3 | 0.004 |
| 2.74_337.1094n | x |  | 1.5 | 0.050 |
| 2.78_112.0752n | x |  | 1.2 | 0.047 |
| 2.78_140.0662n | x |  | 1.3 | 0.068 |
| 2.78_217.1143n | x |  | 1.2 | 0.095 |
| 2.80_166.0970n | x |  | -1.2 | 0.061 |
| 2.84_131.0406n | x |  | -3.9 | 0.015 |
| 2.84_177.0865n | x |  | -3.3 | 0.018 |
| 2.84_178.0164n | x |  | -2.7 | 0.018 |
| 2.84_219.0565n | x |  | -3.4 | 0.016 |
| 2.84_220.0597n | x |  | -3.3 | 0.018 |
| 2.84_281.0269n | x |  | -2.5 | 0.016 |
| 2.84_454.0778n |  |  | -5.3 | 0.019 |
| 2.84_486.1332n |  |  | -1.2 | 0.046 |
| 2.86_241.1181m/z | x |  | -1.3 | 0.035 |
| 2.86_480.1864m/z | x |  | 2.3 | 0.003 |
| 2.88_381.1058n | x |  | 1.6 | 0.013 |
| 2.91_249.0103m/z |  |  | 1.2 | 0.051 |
| 2.91_326.0610n | x |  | 1.6 | 0.051 |
| 2.91_327.0745n |  |  | -1.7 | 0.045 |
| 2.95_174.0433n | x |  | -1.2 | 0.096 |
| 2.97_285.1357m/z | x |  | 1.5 | 0.022 |
| 2.97_299.0496n | x |  | -4.2 | 0.016 |
| 2.97_86.0393n |  |  | 1.2 | 0.090 |
| 3.01_217.0950n | x |  | -1.2 | 0.077 |
| 3.01_461.1682m/z | x |  | 1.7 | 0.002 |
| 3.04_241.1426n |  |  | -1.2 | 0.088 |
| 3.05_353.1684n |  |  | -1.3 | 0.100 |
| 3.10_487.1577n | x |  | 1.7 | 0.048 |
| 3.12_263.1480n |  |  | 1.4 | 0.027 |
| 3.14_165.1135n | x |  | -2.0 | 0.051 |
| 3.14_212.0619n |  |  | -1.3 | 0.067 |
| 3.14_355.0901n | x |  | 1.6 | 0.024 |
| 3.16_175.0664n |  |  | 1.4 | 0.053 |
| 3.22_329.1708n | x |  | -1.2 | 0.094 |
| 3.24_211.0780n | x |  | 2.2 | 0.018 |
| 3.26_291.0702n |  |  | -1.3 | 0.014 |
| 3.26_386.1839n | x |  | 1.8 | 0.003 |
| 3.29_217.1143n | x |  | -1.2 | 0.087 |
| 3.30_274.0912n | x |  | 1.3 | 0.028 |
| 3.30_400.2322n |  |  | 1.3 | 0.079 |
| 3.32_399.1163n | x |  | 1.4 | 0.088 |
| 3.40_420.1717n | x |  | 2.1 | 0.015 |
| 3.43_328.1632n |  |  | -1.3 | 0.088 |
| 3.45_272.0374n |  |  | -1.1 | 0.081 |
| 3.45_274.6120m/z | x |  | 2.3 | 0.024 |
| 3.45_285.1687n | x |  | -1.4 | 0.042 |
| 3.45_642.2630n | x |  | 1.6 | 0.043 |
| 3.51_287.0461n | x |  | 1.3 | 0.085 |
| 3.51_452.1616n | x |  | 2.1 | 0.012 |
| 3.55_273.0310n | x |  | 1.3 | 0.074 |
| 3.57_192.0642m/z | x |  | 2.0 | 0.053 |
| 3.59_244.0075m/z | x |  | 1.4 | 0.098 |
| 3.59_259.0149n | x |  | 1.3 | 0.072 |
| 3.59_395.1552n | x |  | -1.2 | 0.075 |
| 3.61_181.1335m/z | x |  | -1.3 | 0.086 |
| 3.61_531.2147n | x |  | 2.0 | 0.007 |
| 3.63_404.1405n | x |  | 1.6 | 0.026 |
| 3.65_421.1685m/z | x |  | 1.7 | 0.022 |
| 3.67_241.1118n | x |  | 1.5 | 0.034 |
| 3.73_196.0769n | x |  | -1.2 | 0.089 |
| 3.73_316.1203n | x |  | 2.0 | 0.005 |
| 3.75_211.0576m/z | x |  | -1.2 | 0.035 |
| 3.75_416.0453m/z | x |  | 1.4 | 0.060 |
| 3.77_163.0926n |  |  | 1.2 | 0.067 |
| 3.77_184.0713n | x |  | 1.2 | 0.054 |
| 3.77_401.0657n | x |  | 1.4 | 0.035 |
| 3.77_413.0933n | x |  | 1.6 | 0.061 |
| 3.77_480.0979m/z | x |  | 1.4 | 0.058 |
| 3.82_316.1634n |  |  | -1.2 | 0.055 |
| 3.86_563.1640n | x |  | 1.7 | 0.003 |
| 3.88_444.1324n | x |  | 2.3 | 0.001 |
| 3.90_337.0478n | x |  | 6.3 | 0.070 |
| 3.90_421.1483n |  |  | -1.2 | 0.071 |
| 3.90_503.1427n | x |  | 2.3 | 0.009 |
| 3.92_259.0794m/z | x |  | 11.8 | 0.065 |
| 3.96_314.0862n | x |  | 2.1 | 0.005 |
| 4.00_473.1686n | x |  | 1.8 | 0.001 |
| 4.00_475.1751n | x |  | 1.8 | 0.002 |
| 4.02_215.0817n | x |  | 1.9 | 0.003 |
| 4.10_210.1363n | x |  | 1.2 | 0.021 |
| 4.10_343.1735n | x |  | -1.2 | 0.097 |
| 4.21_504.1812n | x |  | 1.5 | 0.028 |
| 4.25_177.0635m/z | x |  | -1.3 | 0.047 |
| 4.29_375.1503n | x |  | 1.5 | 0.020 |
| 4.29_539.1639n | x |  | 2.2 | 0.001 |
| 4.31_263.0105n | x |  | 1.3 | 0.046 |
| 4.31_337.1523n | x |  | 1.9 | 0.016 |
| 4.33_188.0837n | x |  | 1.3 | 0.076 |
| 4.33_275.0462n | x |  | 1.6 | 0.007 |
| 4.35_448.1995n | x |  | 2.1 | 0.006 |
| 4.39_252.0716n | x |  | -1.1 | 0.066 |
| 4.48_506.1927m/z | x |  | 1.7 | 0.009 |
| 4.49_288.1505m/z | x |  | 1.5 | 0.020 |
| 4.54_138.0510n |  |  | -1.3 | 0.087 |
| 4.54_467.0886n | x |  | 1.8 | 0.020 |
| 4.54_548.1395n | x |  | 1.8 | 0.001 |
| 4.55_515.1429n | x |  | 1.9 | 0.029 |
| 4.55_563.1639n | x |  | 1.7 | 0.002 |
| 4.60_394.0981n |  |  | 1.3 | 0.016 |
| 4.60_527.1827n | x |  | 1.3 | 0.096 |
| 4.62_369.1093n | x |  | 1.3 | 0.067 |
| 4.64_159.0846m/z |  |  | -1.3 | 0.039 |
| 4.66_188.1640n |  |  | -2.3 | 0.060 |
| 4.68_307.1993n | x |  | 2.0 | 0.001 |
| 4.68_363.0774n | x |  | 1.5 | 0.031 |
| 4.70_453.0729n | x |  | 1.7 | 0.021 |
| 4.72_361.1734n |  |  | -1.3 | 0.040 |
| 4.72_377.0931n | x |  | 1.5 | 0.069 |
| 4.72_409.1735n | x |  | 1.7 | 0.018 |
| 4.76_600.1620n | x |  | 1.8 | 0.016 |
| 4.78_423.1527n | x |  | 1.5 | 0.029 |
| 4.82_362.1588n | x |  | 1.8 | 0.007 |
| 4.84_269.0632n |  |  | -10.1 | 0.098 |
| 4.84_524.1950m/z | x |  | 1.8 | 0.015 |
| 4.88_323.1941n | x |  | -1.2 | 0.091 |
| 4.90_195.0644n |  |  | -1.5 | 0.057 |
| 4.92_257.1357n | x |  | 2.0 | 0.025 |
| 4.92_335.0824n | x |  | 2.6 | 0.008 |
| 4.94_149.0710n | x |  | 1.8 | 0.000 |
| 5.00_320.1728n | x |  | 1.9 | 0.004 |
| 5.02_440.1430n | x |  | 1.4 | 0.053 |
| 5.06_417.1979m/z |  |  | -1.7 | 0.043 |
| 5.09_368.1359m/z | x |  | 1.3 | 0.074 |
| 5.11_287.0979n | x |  | 1.5 | 0.003 |
| 5.11_532.1835n | x |  | 1.8 | 0.003 |
| 5.15_443.1895n | x |  | 1.3 | 0.034 |
| 5.15_525.1752n | x |  | 1.4 | 0.031 |
| 5.20_440.1431n | x |  | 1.4 | 0.071 |
| 5.22_285.0160n | x |  | -1.1 | 0.039 |
| 5.22_459.2106n | x |  | 1.8 | 0.011 |
| 5.22_569.1748n | x |  | 1.9 | 0.008 |
| 5.24_490.2097n | x |  | 1.8 | 0.002 |
| 5.26_286.1527n |  |  | 1.3 | 0.015 |
| 5.26_409.0977n |  |  | 1.2 | 0.017 |
| 5.28_247.0270m/z | x |  | 1.3 | 0.069 |
| 5.28_418.0931m/z | x |  | 2.6 | 0.002 |
| 5.28_487.1843n | x |  | 1.6 | 0.001 |
| 5.28_522.2241m/z | x |  | 1.6 | 0.003 |
| 5.34_323.1002n | x |  | 1.5 | 0.059 |
| 5.36_485.2253n | x |  | 1.8 | 0.003 |
| 5.38_577.1792n | x |  | 1.9 | 0.000 |
| 5.40_580.1901n | x |  | 1.9 | 0.000 |
| 5.46_238.0388n |  |  | 1.4 | 0.041 |
| 5.49_379.1089n | x |  | 2.0 | 0.001 |
| 5.53_409.1098n | x |  | 1.8 | 0.067 |
| 5.53_467.2149n | x |  | 1.7 | 0.012 |
| 5.53_503.1737n | x |  | 1.4 | 0.023 |
| 5.55_492.2134m/z | x |  | 1.8 | 0.025 |
| 5.57_358.1739n | x |  | 1.4 | 0.033 |
| 5.63_461.1735n | x |  | 1.9 | 0.002 |
| 5.69_287.1179n | x |  | 1.6 | 0.011 |
| 5.69_449.1687n | x |  | 1.8 | 0.008 |
| 5.69_501.1635n | x |  | 1.5 | 0.010 |
| 5.71_317.1473n | x |  | -1.3 | 0.065 |
| 5.71_325.1288n | x |  | 1.6 | 0.005 |
| 5.71_483.3167n |  |  | -2.7 | 0.052 |
| 5.76_377.1473n | x |  | 1.3 | 0.030 |
| 5.76_563.1641n | x |  | 2.1 | 0.000 |
| 5.76_712.1668m/z | x |  | 1.4 | 0.084 |
| 5.78_363.1680n | x |  | 1.7 | 0.010 |
| 5.78_502.1726n | x |  | 2.0 | 0.015 |
| 5.80_275.9979n | x |  | 1.2 | 0.094 |
| 5.80_305.0002m/z | x |  | 1.2 | 0.052 |
| 5.85_467.0887n | x |  | 1.5 | 0.048 |
| 5.85_488.1545n | x |  | 1.2 | 0.079 |
| 5.89_198.0211n |  |  | -1.2 | 0.088 |
| 5.89_244.0259n | x |  | -1.4 | 0.005 |
| 5.89_248.0571n | x |  | -1.3 | 0.032 |
| 5.89_398.1187n | x |  | -1.5 | 0.083 |
| 5.91_307.2016n | x |  | -1.2 | 0.069 |
| 5.95_449.1120n |  |  | 2.2 | 0.013 |
| 5.97_311.1503n | x |  | 1.3 | 0.033 |
| 5.97_522.2240m/z | x |  | 1.7 | 0.004 |
| 5.99_256.1784n | x |  | 2.7 | 0.059 |
| 5.99_353.0180m/z | x |  | 3.4 | 0.071 |
| 5.99_357.9802n | x |  | 3.2 | 0.090 |
| 6.03_280.1997n |  |  | -2.2 | 0.028 |
| 6.03_383.1214n | x |  | 1.8 | 0.009 |
| 6.13_431.2207n | x |  | 1.9 | 0.021 |
| 6.15_240.5535m/z | x |  | 1.7 | 0.006 |
| 6.15_330.1692n | x |  | 1.6 | 0.008 |
| 6.15_385.7294m/z | x |  | -1.3 | 0.094 |
| 6.15_409.1194n | x |  | 1.7 | 0.004 |
| 6.17_311.1483n | x |  | 1.7 | 0.012 |
| 6.17_402.7217m/z | x |  | -3.1 | 0.081 |
| 6.22_483.2104n | x |  | 1.4 | 0.034 |
| 6.24_237.1212n | x |  | 1.4 | 0.048 |
| 6.26_417.1422n | x |  | 1.9 | 0.050 |
| 6.29_285.1960n | x |  | 1.6 | 0.002 |
| 6.31_379.1088n | x |  | 1.9 | 0.007 |
| 6.37_159.0876n | x |  | -1.3 | 0.090 |
| 6.39_346.1468m/z | x |  | 2.0 | 0.023 |
| 6.39_501.1634n | x |  | 1.8 | 0.007 |
| 6.41_120.0211n | x |  | 2.4 | 0.079 |
| 6.41_328.2109n | x |  | -1.2 | 0.100 |
| 6.41_435.0988n | x |  | 2.0 | 0.003 |
| 6.47_357.1313n |  |  | 2.0 | 0.003 |
| 6.49_181.1216n | x |  | 1.9 | 0.086 |
| 6.51_254.0935m/z | x |  | 1.7 | 0.006 |
| 6.56_299.0980n | x |  | 1.9 | 0.018 |
| 6.56_409.0684n | x |  | 1.3 | 0.082 |
| 6.62_210.1276n | x |  | 2.1 | 0.014 |
| 6.62_233.0600n | x |  | 2.2 | 0.016 |
| 6.67_147.0673n | x |  | 1.4 | 0.060 |
| 6.67_267.1139n | x |  | 1.3 | 0.088 |
| 6.67_286.1527n | x |  | 1.3 | 0.033 |
| 6.67_487.1842n | x |  | 2.0 | 0.033 |
| 6.69_385.1103m/z | x |  | -1.3 | 0.049 |
| 6.69_400.2144n | x |  | 2.2 | 0.012 |
| 6.76_258.1941n | x |  | 2.3 | 0.079 |
| 6.76_316.1632n |  |  | -1.2 | 0.071 |
| 6.78_270.1942n | x |  | 2.6 | 0.061 |
| 6.80_275.1496n | x |  | 1.3 | 0.068 |
| 6.82_379.1087n | x |  | 1.4 | 0.080 |
| 6.89_384.1994n |  |  | 2.2 | 0.039 |
| 6.94_282.1057n | x |  | 1.2 | 0.090 |
| 6.94_405.2150n | x |  | 1.8 | 0.010 |
| 6.96_464.1907n | x |  | 3.1 | 0.001 |
| 6.98_115.0450n |  |  | -2.5 | 0.052 |
| 6.98_203.0615n | x |  | -2.1 | 0.072 |
| 7.00_268.0814m/z | x |  | 3.3 | 0.055 |
| 7.00_323.1003n | x |  | 1.4 | 0.079 |
| 7.00_467.0888n | x |  | 2.2 | 0.026 |
| 7.00_495.1830n | x |  | 1.5 | 0.043 |
| 7.05_228.0826n | x |  | 1.9 | 0.008 |
| 7.12_505.1582n | x |  | 1.8 | 0.017 |
| 7.16_483.1531n | x |  | 1.5 | 0.078 |
| 7.18_479.1760n | x |  | 1.4 | 0.098 |
| 7.28_403.1265n | x |  | 1.6 | 0.048 |
| 7.28_429.1473n | x |  | 2.1 | 0.077 |
| 7.30_292.0490m/z |  |  | -1.6 | 0.081 |
| 7.30_442.2050n |  |  | 2.0 | 0.089 |
| 7.34_328.1633n | x |  | -1.2 | 0.086 |
| 7.34_428.2256n |  |  | 2.7 | 0.025 |
| 7.34_567.1924n | x |  | 1.9 | 0.033 |
| 7.39_313.1541n | x |  | 1.5 | 0.030 |
| 7.41_279.2045m/z |  |  | -1.9 | 0.023 |
| 7.41_398.2165n | x |  | 1.9 | 0.034 |
| 7.48_420.2060n |  |  | 1.3 | 0.095 |
| 7.48_484.2261n | x |  | 1.4 | 0.038 |
| 7.59_348.2364n |  |  | -1.6 | 0.042 |
| 7.59_429.1423n | x |  | 1.8 | 0.055 |
| 7.62_346.2222m/z |  |  | -1.4 | 0.024 |
| 7.64_451.1796n | x |  | 1.6 | 0.030 |
| 7.66_273.1938n | x |  | -1.5 | 0.062 |
| 7.71_472.2520n |  |  | 3.2 | 0.027 |
| 7.73_332.2415n |  |  | -1.7 | 0.043 |
| 7.94_247.0080m/z |  |  | 9.9 | 0.007 |
| 8.01_228.1837n | x |  | 1.7 | 0.091 |
| 8.25_575.2403n | x |  | 1.8 | 0.014 |
| 8.34_249.1033n |  |  | 1.3 | 0.016 |
| 8.34_281.1165n | x |  | 3.3 | 0.094 |
| 8.41_495.2833n | x |  | 1.7 | 0.002 |
| 8.53_323.1003n | x |  | 1.5 | 0.027 |
| 8.60_474.1526n |  |  | 1.5 | 0.088 |
| 8.80_226.0516n |  |  | -1.4 | 0.083 |
| 8.80_284.0413n |  |  | -1.3 | 0.050 |
| 8.80_286.0400n |  |  | -1.3 | 0.043 |
| 8.93_286.0874n |  |  | 1.5 | 0.054 |
| 8.96_390.0982n | x |  | -1.2 | 0.069 |
| 9.04_517.1589n | x |  | 2.1 | 0.057 |
| 9.09_295.1888n | x |  | -1.2 | 0.031 |
| 9.48_588.1442m/z | x |  | 3.3 | 0.093 |
| 9.51_549.0709n | x |  | 2.4 | 0.051 |
| 9.55_479.0887n | x |  | 1.9 | 0.016 |
| 9.55_527.0802n | x |  | 1.9 | 0.026 |
| 9.59_422.0669n |  |  | 10.8 | 0.064 |
| 9.59_492.0403n |  |  | 6.4 | 0.022 |
| 9.62_165.9911n |  |  | 4.8 | 0.061 |
| 9.89_443.2341n | x |  | 1.4 | 0.046 |
| 9.93_296.1215n | x |  | -1.3 | 0.071 |

^1^Cut-off criterion for peak/metabolite that differentiates OUD-(p) versus OUD (n) is p<0.1 (t-test). The unidentified or unannotated peaks are listed with retention (RT) and exact mass (m/z or neutral mass). Metabolites/peaks that also differentiated opium user from none user are labeled with “x”. ^b^Ontology levels: OL1, highly confident identification based on matching with In-house physical standard library (IPSL) via retention time (RT, with RT error≤|0.5|), exact mass (MS, with mass error<5ppm), and tandem mass similarity (MS/MS, with similarity ≥30); ^2^ Ontology: OL2a, confident identification based on matching with IPSL via MS and RT; OL2b, annotation for the isomer or derivatives of the compound listed but not the compound itself, based on matching with IPSL via MS and MS/MS; PDa, annotation based on matching with public database via MS and experimental MS/MS (could be the listed compound, or the isomer or derivatives of the listed compound); PDb, annotation based on matching with public database via MS and predict MS/MS; PDc, annotation for the listed compound based on matching with public database via MS and isotopic similarity or adducts; PDd annotation for listed compound based on matching with public database via MS; N/A, signal was not identified or annotated. ^3^FC, fold change, the ratio of intensity between the OUD (p) subjects vs OUD (n), based on the mean, indicates the direction and magnitude of change: FC>1.0 indicates mean OUD (p) > mean OUD (n). ^4^p-value, determined by t-test (assuming unequal variances, Satterthwaite)

**Table S2. Candidate pathways, based on the similarity of m/z using** **Mummichog, that differentiated high opium users diagnosed as OUD positive from high opium users diagnosed as OUD negative.**

| **Pathway Name** | ^1^**Pathway total** | ^2^**Hits.total** | ^3^**Hits.sig** | ^4^**Gamma** | ^5^**Pathway Number** |
| --- | --- | --- | --- | --- | --- |
| Vitamin B9 (folate) metabolism | 33 | 20 | 7 | 0.04 | P0 |
| Purine metabolism | 80 | 55 | 10 | 0.04 | P1 |
| Drug metabolism - cytochrome P450 | 53 | 51 | 9 | 0.04 | P2 |
| Keratan sulfate degradation | 68 | 6 | 3 | 0.04 | P3 |
| N-Glycan Degradation | 16 | 7 | 3 | 0.04 | P4 |
| R Group Synthesis | 7 | 4 | 2 | 0.05 | P5 |
| Vitamin H (biotin) metabolism | 5 | 4 | 2 | 0.05 | P6 |
| Fatty acid oxidation, peroxisome | 28 | 6 | 2 | 0.06 | P7 |
| Glycosphingolipid metabolism | 67 | 28 | 4 | 0.06 | P8 |
| Fatty acid oxidation | 35 | 7 | 2 | 0.06 | P9 |
| Aminosugars metabolism | 69 | 32 | 4 | 0.07 | P10 |
| Phosphatidylinositol phosphate metabolism | 59 | 22 | 3 | 0.07 | P11 |
| Glycosphingolipid biosynthesis - ganglioseries | 62 | 10 | 2 | 0.08 | P12 |
| Vitamin B5 - CoA biosynthesis from pantothenate | 12 | 11 | 2 | 0.08 | P13 |
| De novo fatty acid biosynthesis | 106 | 26 | 3 | 0.09 | P14 |
| Selenoamino acid metabolism | 35 | 14 | 2 | 0.09 | P15 |
| Sialic acid metabolism | 107 | 30 | 3 | 0.10 | P16 |
| Vitamin A (retinol) metabolism | 67 | 32 | 3 | 0.11 | P17 |
| Methionine and cysteine metabolism | 94 | 48 | 4 | 0.12 | P18 |
| Carnitine shuttle | 72 | 19 | 2 | 0.12 | P19 |
| D4&E4-neuroprostanes formation | 37 | 19 | 2 | 0.12 | P20 |
| Urea cycle/amino group metabolism | 85 | 50 | 4 | 0.12 | P21 |
| Hexose phosphorylation | 20 | 20 | 2 | 0.12 | P22 |
| Fatty acid activation | 74 | 22 | 2 | 0.13 | P23 |
| Histidine metabolism | 33 | 24 | 2 | 0.14 | P24 |
| Tryptophan metabolism | 94 | 69 | 4 | 0.19 | P25 |
| Valine, leucine and isoleucine degradation | 65 | 35 | 2 | 0.19 | P26 |
| Tyrosine metabolism | 160 | 88 | 5 | 0.20 | P27 |
| Pyrimidine metabolism | 70 | 45 | 2 | 0.24 | P28 |
| Aspartate and asparagine metabolism | 114 | 69 | 3 | 0.25 | P29 |
| C21-steroid hormone biosynthesis and metabolism | 112 | 77 | 3 | 0.28 | P30 |
| Androgen and estrogen biosynthesis and metabolism | 95 | 69 | 2 | 0.34 | P31 |
| Xenobiotics metabolism | 110 | 80 | 2 | 0.39 | P32 |
| Hyaluronan Metabolism | 8 | 2 | 1 | 1.00 | P33 |
| N-Glycan biosynthesis | 48 | 13 | 1 | 1.00 | P34 |
| Chondroitin sulfate degradation | 37 | 5 | 1 | 1.00 | P35 |
| Linoleate metabolism | 46 | 20 | 1 | 1.00 | P36 |
| Galactose metabolism | 41 | 35 | 1 | 1.00 | P37 |
| Alkaloid biosynthesis II | 10 | 7 | 1 | 1.00 | P38 |
| Glycosphingolipid biosynthesis - globoseries | 16 | 5 | 1 | 1.00 | P39 |
| Glycosylphosphatidylinositol (GPI)-anchor biosynthesis | 6 | 3 | 1 | 1.00 | P40 |
| Parathio degradation | 6 | 5 | 1 | 1.00 | P41 |
| Biopterin metabolism | 22 | 14 | 1 | 1.00 | P42 |
| Vitamin E metabolism | 54 | 25 | 1 | 1.00 | P43 |
| Glycine, serine, alanine and threonine metabolism | 88 | 52 | 1 | 1.00 | P44 |
| Fatty Acid Metabolism | 63 | 19 | 1 | 1.00 | P45 |
| Saturated fatty acids beta-oxidation | 36 | 19 | 1 | 1.00 | P46 |
| CoA Catabolism | 7 | 6 | 1 | 1.00 | P47 |
| Butanoate metabolism | 34 | 27 | 1 | 1.00 | P48 |
| Ascorbate (Vitamin C) and Aldarate Metabolism | 29 | 20 | 1 | 1.00 | P49 |
| Heparan sulfate degradation | 34 | 6 | 1 | 1.00 | P50 |
| Polyunsaturated fatty acid biosynthesis | 21 | 9 | 1 | 1.00 | P51 |
| Alanine and Aspartate Metabolism | 30 | 22 | 1 | 1.00 | P52 |
| Glutathione Metabolism | 19 | 13 | 1 | 1.00 | P53 |
| Limonene and pinene degradation | 10 | 6 | 1 | 1.00 | P54 |
| Arginine and Proline Metabolism | 45 | 37 | 1 | 1.00 | P55 |
| Glutamate metabolism | 15 | 12 | 1 | 1.00 | P56 |
| Porphyrin metabolism | 43 | 23 | 1 | 1.00 | P57 |
| Prostaglandin formation from arachidonate | 78 | 61 | 1 | 1.00 | P58 |
| Leukotriene metabolism | 92 | 47 | 1 | 1.00 | P59 |
| Vitamin B12 (cyanocobalamin) metabolism | 9 | 5 | 1 | 1.00 | P60 |
| Drug metabolism - other enzymes | 31 | 26 | 1 | 1.00 | P61 |
| Phytanic acid peroxisomal oxidation | 34 | 12 | 1 | 1.00 | P62 |
| Pentose phosphate pathway | 37 | 34 | 1 | 1.00 | P63 |
| Lysine metabolism | 52 | 30 | 1 | 1.00 | P64 |

^1^Pathway total indicates the overall number of metabolites that are included in a specific pathway ^2^Hits.total indicates the number of measured signals that are matched (m/z error<3 ppm) with the metabolites included in the pathway; ^3^Hits.sig indicates the number of matched signals that were significantly changed between phenotypic groups; ^4^Gamma is an adjusted Fisher’s p-value (null distribution) calculated after permutations to determine the significance of the enriched pathway [1] in Metaboanalyst; ^5^Pathways Number listed in Table S2 corresponds to Figure 2 in the main text.

**Table S3. Logistic Modeling Results for Model 1 including demographics and untargeted metabolomics data (712 peaks that differentiated high opium users diagnosed as OUD positive from high opium users diagnosed as OUD negative were used as potential predictors)**

| Model Parameter | Ontology | Compound | Odds Ratio | Lower 95% Confidence Limit | Upper 95% Confidence Limit |
| --- | --- | --- | --- | --- | --- |
|  |  |  |  |  |  |
| Age at Enrollment |  |  | 0.2 | 0.1 | 0.4 |
| Ingestion vs Inhalation |  |  | 0.3 | 0.1 | 1.0 |
| 6.15_385.7294m/z |  |  | 0.4 | 0.3 | 0.7 |
| 13.77_257.2262m/z |  |  | 0.2 | 0.1 | 0.5 |
| 5.11_287.0979n |  |  | 2.5 | 1.2 | 5.1 |
| 10.71_360.1838m/z |  |  | 6.1 | 2.6 | 14.6 |
| 1.91_196.0406n |  |  | 0.4 | 0.2 | 0.9 |
| 7.34_328.1633n |  |  | 0.2 | 0.1 | 0.5 |
| 4.16_230.1022m/z |  |  | 4.1 | 1.6 | 10.4 |
| 7.37_204.0893n | OL2B | Tryptophan | 0.3 | 0.1 | 0.7 |
| 4.88_187.1077m/z |  |  | 0.5 | 0.3 | 0.9 |
| 1.97_164.0567m/z | OL1 | Pterine | 3.2 | 1.7 | 6.0 |
| 4.66_188.1640n |  |  | 0.2 | 0.0 | 0.6 |
| 5.26_409.0977n |  |  | 4.2 | 2.1 | 8.4 |
| 11.45_304.1672n |  |  | 4.5 | 1.9 | 10.9 |
| 3.73_180.0892n |  |  | 2.4 | 1.1 | 5.3 |
| 5.89_358.1263n |  |  | 0.2 | 0.0 | 0.5 |
| 4.25_177.0635m/z |  |  | 0.3 | 0.2 | 0.5 |

**Table S4. Logistic Modeling Results for Model 2 including demographics and untargeted metabolomics data (40 identified/annotated peaks that differentiated high opium users diagnosed as OUD positive from high opium users diagnosed as OUD negative were used as potential predictors)**

| Model Parameter | Ontology | Compound | Odds Ratio | Lower 95% Confidence Limit | Upper 95% Confidence  Limit |
| --- | --- | --- | --- | --- | --- |
|  |  |  |  |  |  |
| Age at Enrollment |  |  | 0.6 | 0.4 | 0.8 |
| Ingestion vs Inhalation |  |  | 1.0 | 0.4 | 2.1 |
| 7.37_204.0893n | OL2B | Tryptophan | 0.6 | 0.4 | 0.9 |
| 1.97_164.0567m/z | OL1 | Pterine | 1.7 | 1.2 | 2.6 |
| 0.62_89.0477n | OL1 | Sarcosine | 1.9 | 1.3 | 2.9 |
| 4.64_157.0739n | OL2B | N-Acetylproline | 0.6 | 0.4 | 0.9 |
| 1.37_163.1230m/z | OL1 | Nicotine | 1.7 | 1.1 | 2.6 |
| 8.80_188.1049n | OL1 | Azelate | 0.7 | 0.5 | 1.0 |
| 1.89_153.0785n | OL2B | P-Octopamine | 1.6 | 1.0 | 2.5 |
| 2.84_88.0394m/z | OL2B | L-Serine | 0.4 | 0.2 | 0.8 |

**Table S5. Logistic Modeling Results for Model 3 including demographics and untargeted metabolomics data (14 identified/annotated metabolites that were unique to the differentiation of high opium users diagnosed as OUD positive from high opium users diagnosed as OUD negative (but did not also differentiate opium users from non-opium users) were used as potential predictors)**

| Model Parameter | Ontology | Compound | Odds Ratio | Lower 95% Confidence Limit | Upper 95% Confidence  Limit |
| --- | --- | --- | --- | --- | --- |
| Age at Enrollment |  |  | 0.6 | 0.4 | 0.8 |
| Ingestion vs Inhalation |  |  | 1.8 | 0.9 | 3.6 |
| 3.49_234.0794m/z | OL2A | N-Acetyl-S- (3,4-dihydroxybutyl)-L-cysteine | 0.5 | 0.3 | 0.9 |
| 7.37_204.0893n | OL2B | Tryptophan | 0.7 | 0.5 | 1.0 |
| 1.97_164.0567m/z | OL1 | Pterine | 1.6 | 1.1 | 2.3 |
| 0.62_89.0477n | OL1 | Sarcosine | 1.6 | 1.1 | 2.3 |
| 8.80_188.1049n | OL1 | Azelate | 0.7 | 0.5 | 1.0 |

**Figure S1.** **Comparison of AUC for candidate peaks/metabolites and subject characteristic covariates. Logistic regression models using a) 712 peaks that differentiate high opium users diagnosed as OUD positive verse high opium users diagnosed as OUD negative, b) 40 metabolites that differentiate high opium users diagnosed as OUD positive versus high opium users diagnosed as OUD negative, or c) 14 metabolites unique to the diagnosis of OUD.**


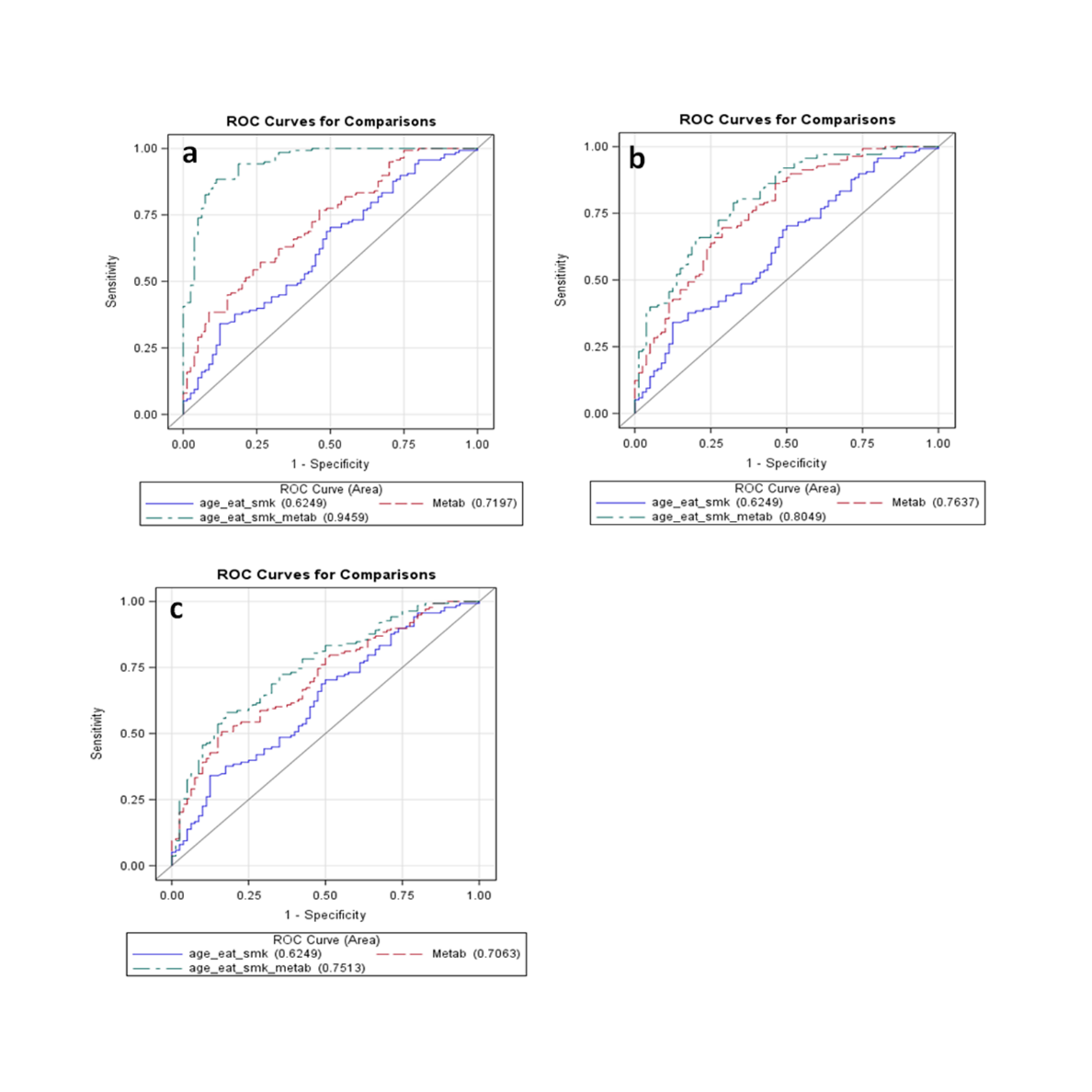


References

1. Li, S. et al., Predicting network activity from high throughput metabolomics. PLoS Comput Biol 9, e1003123 (2013).
