# Supplementary figures and images for: Metabolomics reveals biomarkers of opioid use disorder
Source: Transl Psychiatry. 2021 Feb 4;11:103. doi: 10.1038/s41398-021-01228-7 (PMC7862627; doi:10.1038/s41398-021-01228-7)

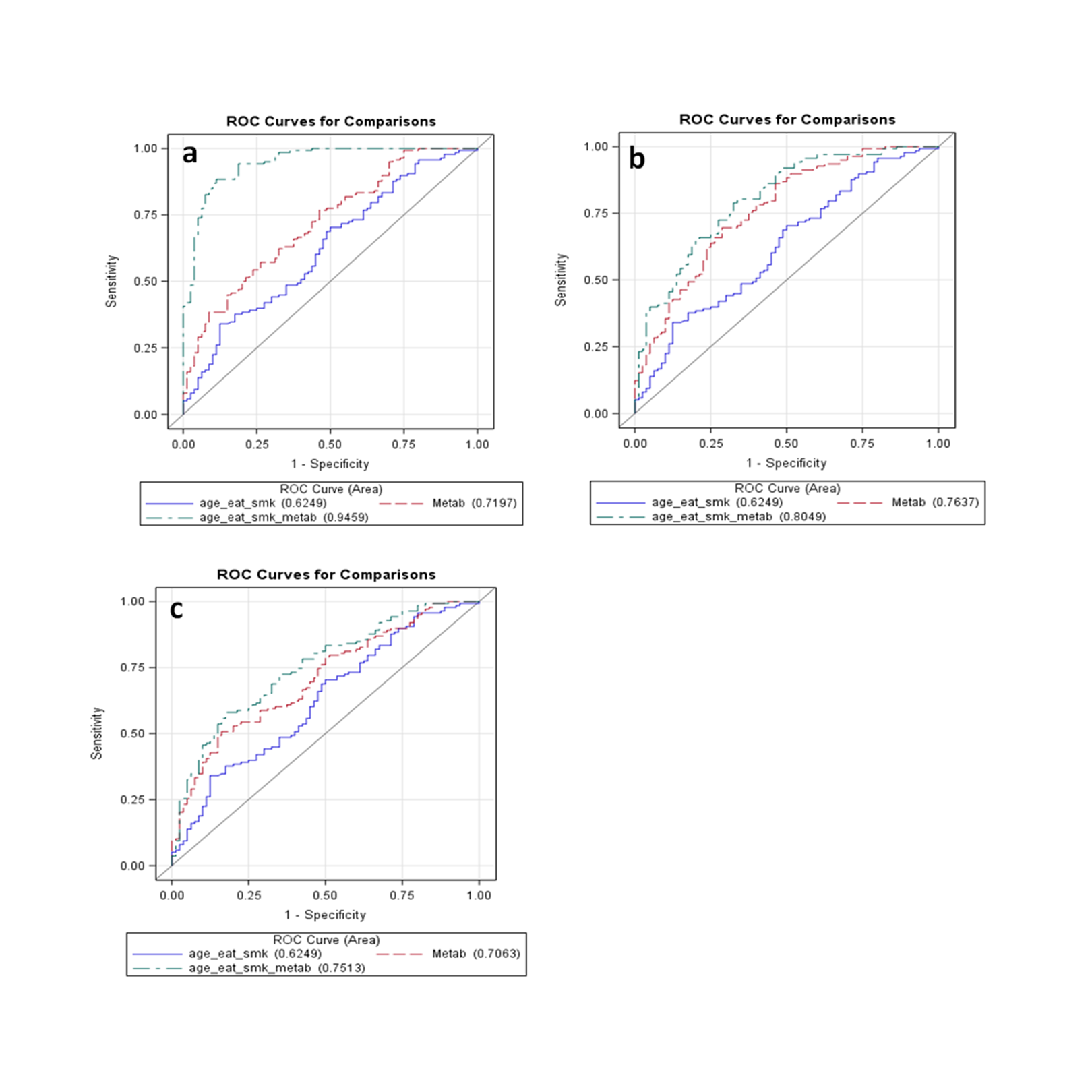

Supplement: Supplementary file 2 — Figure S1 [file 41398_2021_1228_MOESM2_ESM.tif]
